# S1PR1 regulates lymphatic valve development and tertiary lymphoid organ formation in the ileum

**DOI:** 10.1084/jem.20241799

**Published:** 2025-06-24

**Authors:** Xin Geng, Lijuan Chen, Zoheb Ahmed, Guilherme Pedron Formigari, Yen-Chun Ho, Ilaria Del Gaudio, Marcella Neves Datilo, Zheila J. Azartash-Namin, Coraline Heron, Xindi Shan, Ravi Shankar Keshari, Soumiya Pal, Hong Chen, Florea Lupu, Lijun Xia, Gwendalyn J. Randolph, Scott D. Zawieja, Eric Camerer, Michael J. Davis, R. Sathish Srinivasan

**Affiliations:** 1 Cardiovascular Biology Research Program, Oklahoma Medical Research Foundation, Oklahoma City, OK, USA; 2 Université Paris Cité, Inserm, Paris Research Center-Cardiovascular (PARCC), Paris, France; 3 Department of Medical Pharmacology and Physiology, University of Missouri, Columbia, MO, USA; 4 Vascular Biology Program, Boston Children’s Hospital, Boston, MA, USA; 5 Department of Pathology and Immunology, Washington University School of Medicine, St. Louis, MO, USA; 6 Department of Cell Biology, University of Oklahoma Health Sciences Center, Oklahoma City, OK, USA

## Abstract

Efficient lymph flow is ensured by lymphatic valves (LVs). The mechanisms that regulate LV development are incompletely understood. Here, we show that the deletion of the GPCR sphingosine 1-phosphate receptor-1 (S1PR1) from lymphatic endothelial cells (LECs) results in fewer LVs. Interestingly, LVs that remained in the terminal ileum-draining lymphatic vessels were specifically dysfunctional. Furthermore, tertiary lymphoid organs (TLOs) formed in the terminal ileum of the mutant mice. TLOs in this location are associated with ileitis in humans and mice. However, mice lacking S1PR1 did not develop obvious characteristics of ileitis. Mechanistically, S1PR1 regulates shear stress signaling and the expression of the valve-regulatory molecules FOXC2 and connexin-37. Importantly, *Foxc2*^*+/−*^ mice, a model for lymphedema-distichiasis syndrome, also develop TLOs in the terminal ileum. Thus, we have discovered S1PR1 as a previously unknown regulator of LV and TLO development. We also suggest that TLOs are a sign of subclinical inflammation that can form due to lymphatic disorders in the absence of ileitis.

## Teaser

TLOs are LN-like structures that are observed in certain inflammatory diseases. Geng et al. demonstrate that S1PR1 activity in the lymphatic vasculature is necessary to prevent TLO formation in the terminal ileum.

## Graphical Abstract

**Figure:**
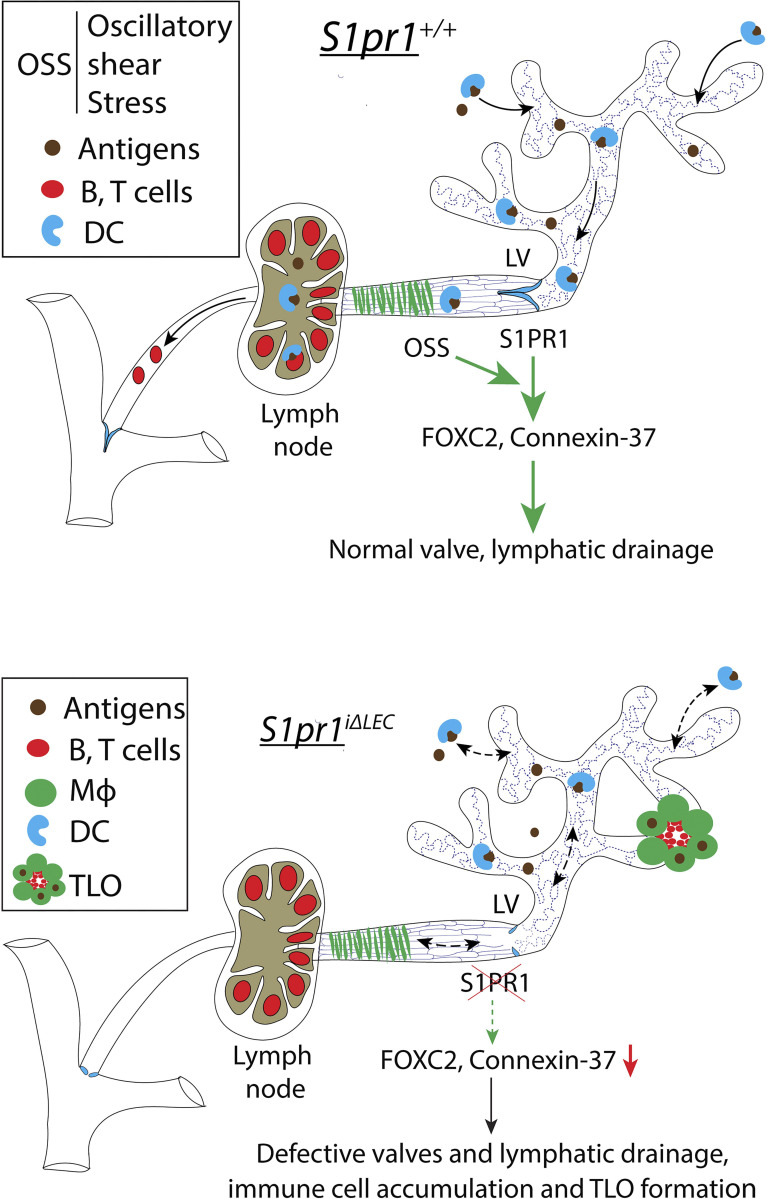

## Introduction

Tertiary lymphoid structures, also known as tertiary lymphoid organs (TLOs), are LN-like structures that form under pathological conditions such as in certain tumors, rejected transplant tissues, lungs of chronic obstructive pulmonary disease or influenza-infected patients, adipose tissue of obese individuals, and in tissues afflicted with autoimmune diseases such as Hashimoto thyroiditis, type 1 diabetes, multiple sclerosis, rheumatoid arthritis, and inflammatory bowel disease (IBD) (Ruddle, 2014). TLOs contain B cells, T cells, macrophages, stromal cells such as fibroblastic reticular cells (FRCs) and follicular dendritic cells, and high endothelial venules (HEVs). Infectious agents such as *Helicobacter pylori*, *Helicobacter hepaticus*, *Borrelia burgdorferi*, and *Salmonella enterica* can also promote TLO development (Overacre-Delgoffe et al., 2021; Drayton et al., 2006, Koscsó et al., 2020). TLOs are thought to function as sites of localized immune response against microbes and parasites, tumor antigens, grafted tissues, and auto antigens. The presence of TLOs is associated with better prognosis in the context of tumor progression and immune checkpoint therapy (Petitprez et al., 2020; Cabrita et al., 2020; Helmink et al., 2020; Schumacher and Thommen, 2022; Overacre-Delgoffe et al., 2021). In contrast, TLOs are thought to aggravate tissue damage in autoimmune diseases and contribute to transplanted tissue rejection (Drayton et al., 2006; Pipi et al., 2018; Nasr et al., 2007; Czepielewski et al., 2021; Randolph et al., 2016). TLOs can progress into lymphomas, as in the context of *Helicobacter pylori* infections (Wroblewski et al., 2010; Violeta Filip et al., 2018). TLOs that develop in adipose tissues during obesity can reduce lipolysis and insulin sensitivity (Camell et al., 2019; Cao et al., 2021). Thus, modulation of TLO formation may be beneficial under various pathological conditions. To achieve such modulation, it is critical to obtain a better understanding of how they form.

TLOs share structural similarities with secondary lymphoid organs such as LNs (Ruddle, 2014). LN development is initiated by the extravasation of hematopoietic-derived lymphoid tissue initiator (LTi) cells at specialized vein junctions that are devoid of smooth muscle cell coverage (Bovay et al., 2018). Nearby lymphatic vessels undergo a cup-shaped morphogenesis to collect LTi and nearby stromal cells, known as lymphoid tissue organizer (LTo) cells. Cross talk between LTi, LTo, and lymphatic endothelial cells (LECs) promotes the organization and expansion of LN primordia. LNs do not develop properly in mice with defective lymphatic vessels (Bovay et al., 2018; Vondenhoff et al., 2009; Onder et al., 2017; Lee and Koh, 2016). Naïve T and B cells enter the mature LNs through HEVs and reside in specific compartments. Afferent lymphatic vessels secrete chemokines, such as CCL21, to recruit antigen-loaded DCs from peripheral tissues, which are then transported to LNs, where they encounter and activate naïve T and B cells. Efferent lymphatic vessels secrete sphingosine 1-phosphate (S1P) to promote the exit of lymphocytes from the LNs into the lymphatic vessels and then into blood (Matloubian et al., 2004; Pham et al., 2010; Pham et al., 2008). Thus, lymphatic vessels are an integral part of LN development and function.

TLOs appear to be heterogeneous with respect to the presence or absence of lymphatic vessels. TLOs in adipose tissue were reported to lack lymphatic vessels (Bénézech et al., 2015), and TLOs in the lungs developed in the absence of lymphatic vessels or lymphatic drainage (Reed et al., 2019). However, more recently, TLOs that were connected to dysfunctional mesenteric lymphatic vessels were identified in high-fat diet-fed mice and in *Tnf*^*+/ΔARE*^ mice (a model for ileitis) (Czepielewski et al., 2021; Cao et al., 2021; Rehal and von der Weid, 2017). These publications demonstrated that chronic inflammation can result in TLO formation. Despite these findings, the relationship between the lymphatic vasculature and TLOs is not fully understood. Whether lymphatic dysfunction can result in TLO formation in the absence of chronic inflammation is also not known.

The lymphatic vasculature consists of lymphatic capillaries, collecting lymphatic vessels, lymphatic valves (LVs), and lymphovenous valves (LVVs) (Geng et al., 2017). LVVs, LVs, and venous valves are structurally alike, similar to each other, and express a similar set of regulatory genes (Srinivasan and Oliver, 2011; Geng et al., 2016; Geng et al., 2017; Bazigou et al., 2011). Vascular valves develop in a stepwise process that involves sensing of oscillatory shear stress (OSS), delamination of cells, cell elongation, and collective cell migration (Geng et al., 2016; Geng et al., 2017; Sabine et al., 2012; Pujol et al., 2017; Tatin et al., 2013; Kazenwadel et al., 2015; Cha et al., 2016; Choi et al., 2019). The G-protein coupled receptor (GPCR) S1P receptor-1 (S1PR1) is an important therapeutic target. Inhibitors of S1PR1 are used to treat inflammatory diseases such as multiple sclerosis and IBD, which are diseases that are likely modulated by the lymphatic vasculature (Cartier and Hla, 2019; Oliver et al., 2020). We recently identified S1PR1 as a regulator of LEC junctions, cytoskeleton, and lymphangiogenesis (Geng et al., 2020). In this study, we tested whether S1PR1 regulated LV and LVV development and maintenance and serendipitously discovered a previously unknown relationship between S1PR1 signaling, LV development, and TLO formation.

## Results

### S1PR1 regulates the development of embryonic LVs, LVVs, and venous valves

**Figure 1.**
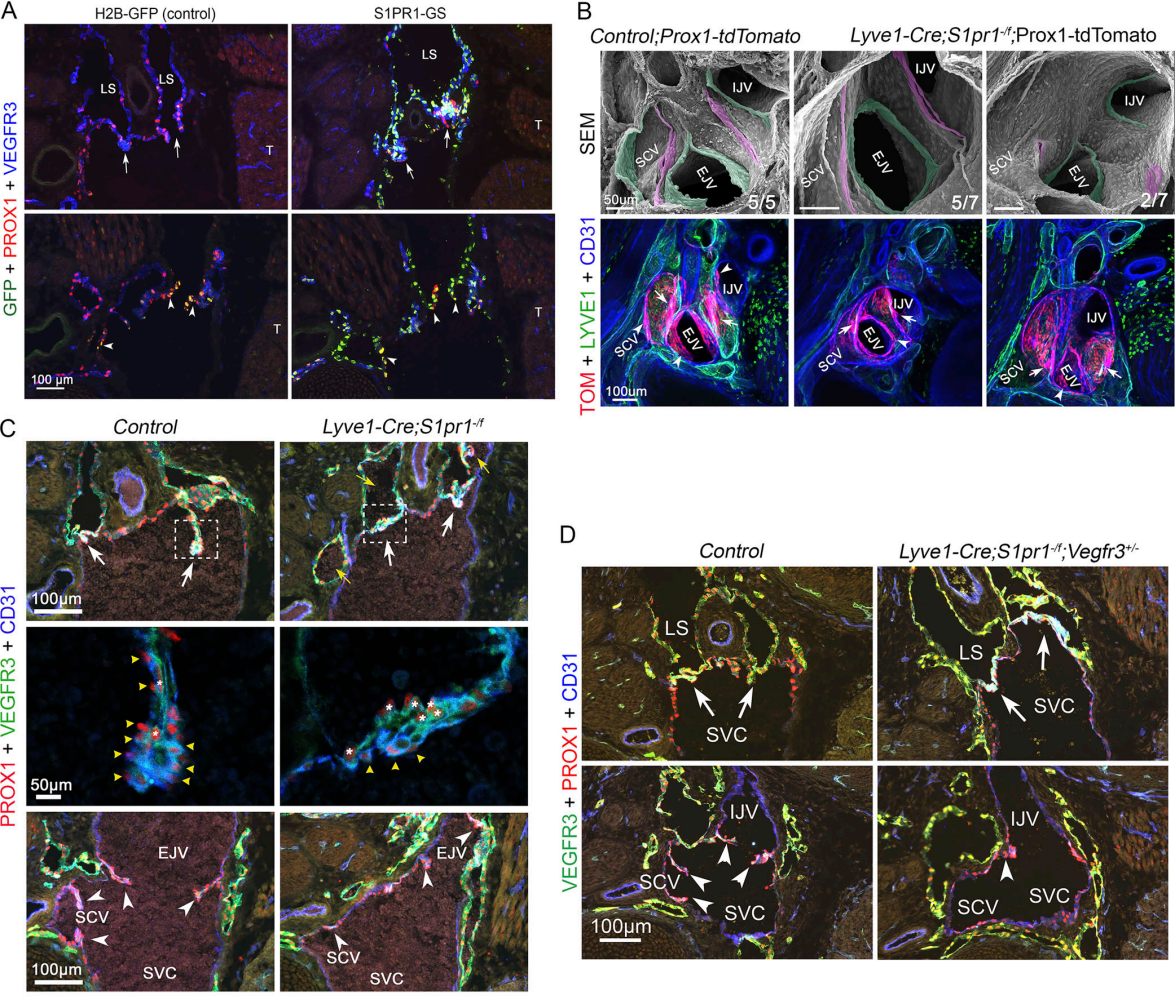
**S1PR1 signaling is active during and is necessary for LVV and venous valve morphogenesis. (A)** E16.5 S1PR1-GS and H2B-GFP (control) littermates were frontally sectioned and analyzed. GFP^+^ cells were observed in the LVVs (arrows) and venous valves (arrowheads) of S1PR1-GS embryos, indicating that S1PR1 signaling is active in these structures. Only a few GFP^+^ cells were observed in H2B-GFP embryos due to leaky expression of this transgene. **(B)** 500-μm–thick transverse section of E16.5 Prox1-tdTomato and *Lyve1-Cre;S1pr1*^*−/f*^; Prox1-tdTomato embryos were prepared using a vibratome, and whole-mount IHC and confocal imaging were performed to visualize the LVVs and venous valves (bottom row). Subsequently, the same samples were processed and analyzed by SEM (top row). Normal-looking LVVs (arrows or pseudo colored in magenta) and venous valves (arrowheads or pseudo colored in green) were seen in control embryos. Mutant embryos appeared to have substantially fewer valvular endothelial cells. When present the mutant cells appeared disorganized and arrested at the periphery of the vessels. **(C and D)** E16.5 control, *Lyve1-Cre;S1pr1*^*−/f*^, and *Lyve1-Cre;S1pr1*^*−/f*^;*Vegfr3*^*+/−*^ embryos were frontally sectioned (12-μm thick) and analyzed by IHC. LVVs (arrows) and venous valves (arrowheads) of control embryos had invaginated into the veins. Higher magnification images of control LVVs (boxed areas and the panels below in C) revealed that VEGFR3^hi^;Prox1^hi^ LECs (asterisks) were located in between two layers of VEGFR3^Low^;Prox1^hi^ valvular endothelial cells (yellow arrowheads). In contrast, invagination was substantially reduced in the LVVs and venous valves of mutant embryos, and the organization of the two cell types was defective. Blood cells were observed within the lymph sacs of mutant embryos (yellow arrows in C). **(D)** The invagination defect of *Lyve1-Cre;S1pr1*^*−/f*^ embryos was not rescued by *Vegfr3* heterozygosity. Statistics: (A, C, and D) *n* = 5 embryos per genotype; (B) *n* = 5 controls and *n* = 7 *Lyve1-Cre;S1pr1*^*−/f*^ LVV complexes. LS, lymph sacs; T, thymus; EJV, external jugular vein; SVC, superior vena cava; SCV, subclavian vein; IJV, internal jugular vein.

Two pairs of LVVs are bilaterally located at the junction of jugular and subclavian veins to regulate lymph return to the blood circulation (Geng et al., 2016; Srinivasan and Oliver, 2011). LVVs are the first vascular valves to develop in mammals. We used the S1PR1 activity reporter mice (“tango” mice, henceforth referred to as S1PR1-GS mice) to test if S1PR1 signaling is active in LVVs (Kono et al., 2014). The S1PR1-GS are double transgenic mice. One knock-in allele at the S1PR1 locus expresses two modified proteins: (1) S1PR1 C-terminally fused to a tetracycline transactivator (tTA), separated by a TEV protease recognition site, and (2) a β-arrestin–TEV protease fusion protein. In the presence of S1P ligand, β-arrestin–TEV protease is recruited to and cleaves the S1PR1-tTA chimeric receptor. Free tTA translocates to the nucleus to activate expression of a tetracycline response element-driven H2B-EGFP reporter from the second transgene. Overall, in S1PR1-GS mice, the interaction of S1PR1 with its ligand S1P and the subsequent β-arrestin coupling to the receptor results in the nuclear expression of GFP. We have used mice with H2B-EGFP reporter alone as controls to differentiate the cells with authentic S1PR1 signaling from cells with leaky expression of the H2B-EGFP reporter. By analyzing embryonic day (E) 16.5 S1PR1-GS embryos, we determined that S1PR1 signaling was indeed active in LVVs (Fig. 1 A, arrows). S1PR1 activity was also observed in the nearby venous valves of the jugular and subclavian veins (Fig. 1 A, arrowheads).

**Figure S1.**
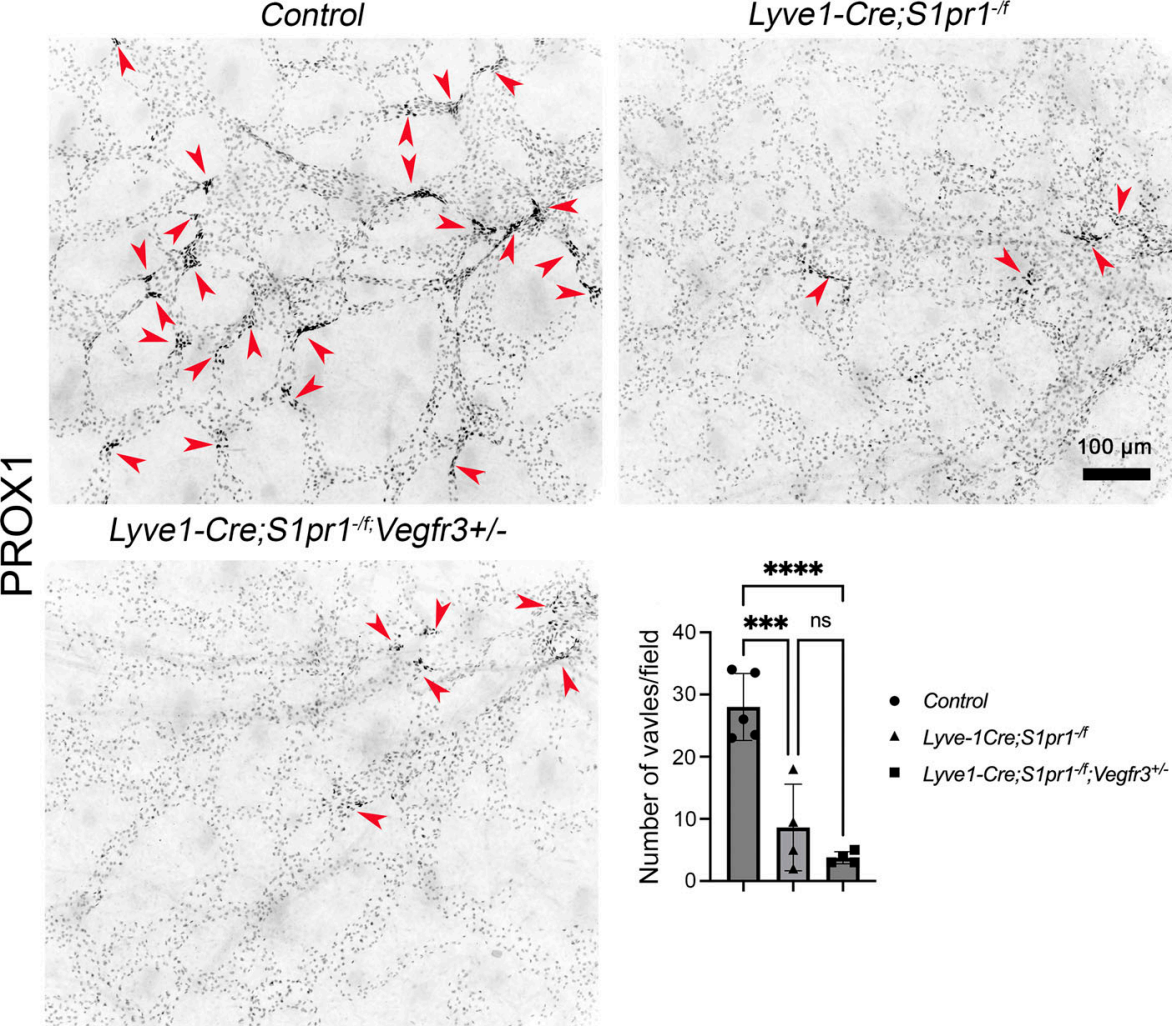
**S1PR1 regulates dermal LV development in a VEGFR3-independent manner.** The dorsal skin of E16.5 embryos was dissected and analyzed by whole-mount IHC with anti-PROX1 antibody. The developing LVs were identified by the presence of PROX1^hi^ clusters (arrowheads). Statistics: *n* = 5 for control embryos; *n* = 4 for each mutant genotype, respectively. Each dot represents one animal on the graph. The graphs were plotted as mean ± SD. One-way ANOVA was performed for the statistical analysis. ***P < 0.001; ****P < 0.0001.

We used *Lyve1-Cre* to delete *S1pr1* from embryos (Geng et al., 2020; Pham et al., 2010). *Lyve1-Cre* is active in the jugular vein and the developing LECs (Cha et al., 2016; Geng et al., 2016). Fluorescent/SEM correlative microscopy revealed that the LVV and venous valve leaflets of E16.5 *Lyve1-Cre; S1pr1*^*−/f*^ embryos were either absent or much shorter than those of the control valves (Fig. 1 B). Immunohistochemistry (IHC) performed on cryosections further revealed that the valvular endothelial cells were specified but failed to organize and invaginate into the veins (Fig. 1 C). Additionally, fewer LVs were observed in the dermal lymphatic vessels of *Lyve1-Cre; S1pr1*^*−/f*^ embryos when compared with control littermates (Fig. S1). Thus, S1PR1 is necessary for the development of LVVs, LVs, and venous valves.

We previously showed that the deletion of *S1pr1* from LECs resulted in excessive lymphatic vessel branching in embryonic skin and that this phenotype can be ameliorated by deleting one allele of *Vegfr3* (Geng et al., 2020). To determine the effect on dermal LV defects in S1PR1 mutants, we analyzed E16.5 *Lyve1-Cre; S1pr1*^*−/f*^; *Vegfr3*^*+/−*^ embryos and determined that the dermal LV defects of S1PR1 mutants were not rescued by *Vegfr3* heterozygosity (Fig. S1). The LVV and venous valve defects of S1PR1 mutants were also not rescued by *Vegfr3* heterozygosity (Fig. 1 D).

Together these data revealed that S1PR1 regulates the development of LVs, LVVs, and venous valves in a *Vegfr3*-independent manner.

### S1PR1 regulates the postnatal development of LVs and maintains the functioning of LVs in the ileum-draining lymphatic vessels

**Figure 2.**
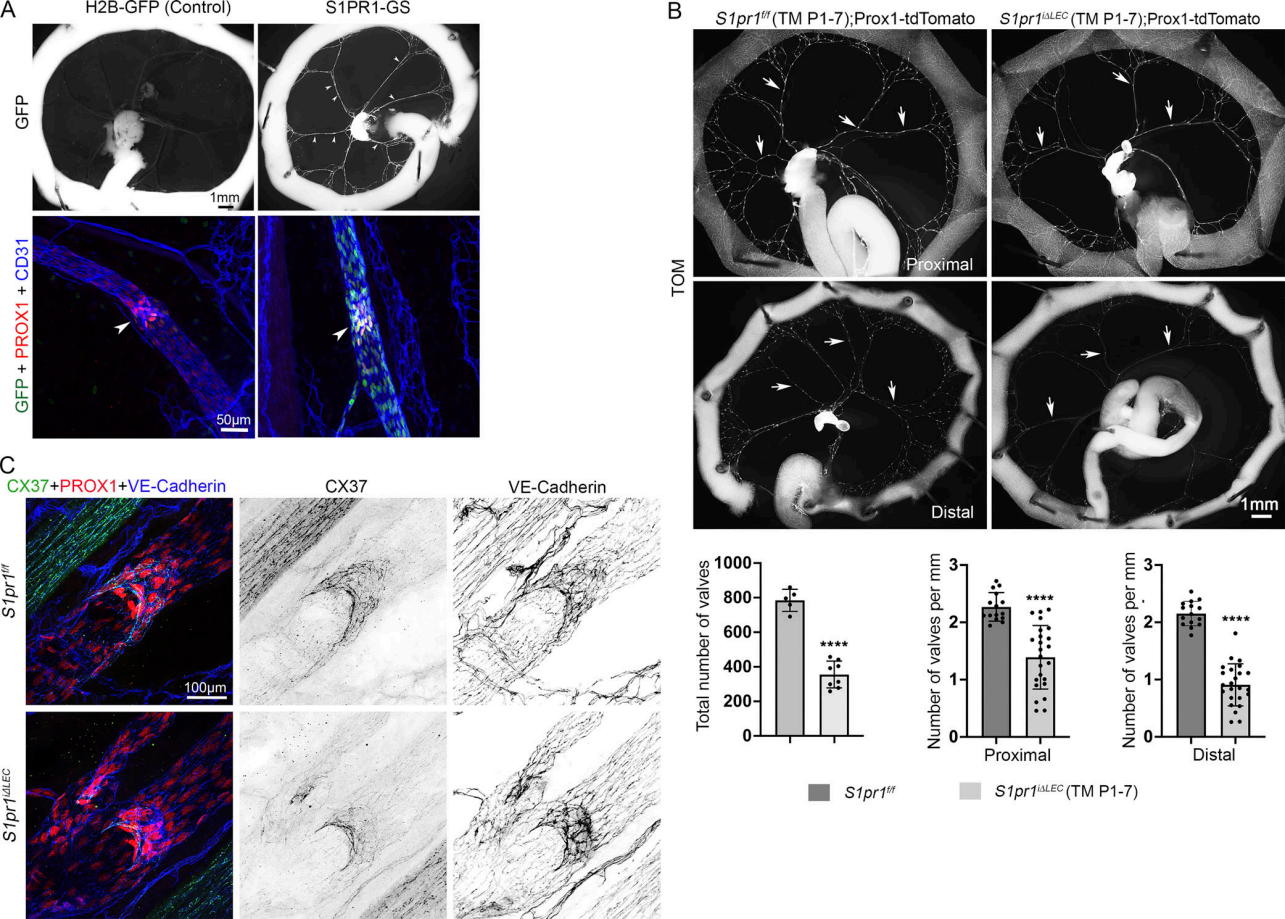
**S1PR1 signaling is active in LVs and is necessary for LV development. (A)** The mesenteries of P10 S1PR1-GFP and H2B-GFP littermates where analyzed. Collecting lymphatic vessels of S1PR1-GFP pups were GFP^+^ and GFP expression was stronger in the LVs (arrowheads). GFP expression was not observed in H2B-GFP pups. **(B)** The mesenteries of P10 *S1pr1*^*f/f*^;Prox1-tdTomato and *S1pr1*^*iΔLEC*^;Prox1-tdTomato pups that were administered TM from P1–7 were analyzed. Mesenteric tissue that was connected to the duodenum and jejunum was considered “proximal,” and that which is connected to the ileum and cecum was considered “distal,” LVs (puncta) in the proximal and distal vessels were counted and quantified. LVs were significantly reduced in *S1pr1*^*iΔLEC*^;Prox1-tdTomato pups. The reduction appeared to be more severe in the posterior section of the gut. **(C)** The mesenteric lymphatic vessels and LVs of P10 pups that were administered TM from P1–7 were analyzed using the indicated antibodies. CX37 expression appeared to be reduced in the remaining LVs of *S1pr1*^*iΔLEC*^ pups. Statistics: (A) *n* = 9 S1PR1-GS and *n* = 3 H2B-GFP pups; (B) *n* = 5 control and *n* = 8 *S1pr1*^*iΔLEC*^ pups. Each dot represents an individual animal on the graph for the total number of LVs. Three proximal vessels and three distal vessels from each mesentery were analyzed to quantify the valve density. Each dot represents a vessel on the graph; (C) *n* = 3 per genotype. The graphs were plotted as mean ± SD. Unpaired *t* test was performed for the statistical analysis. ****P < 0.0001.

**Figure S2.**
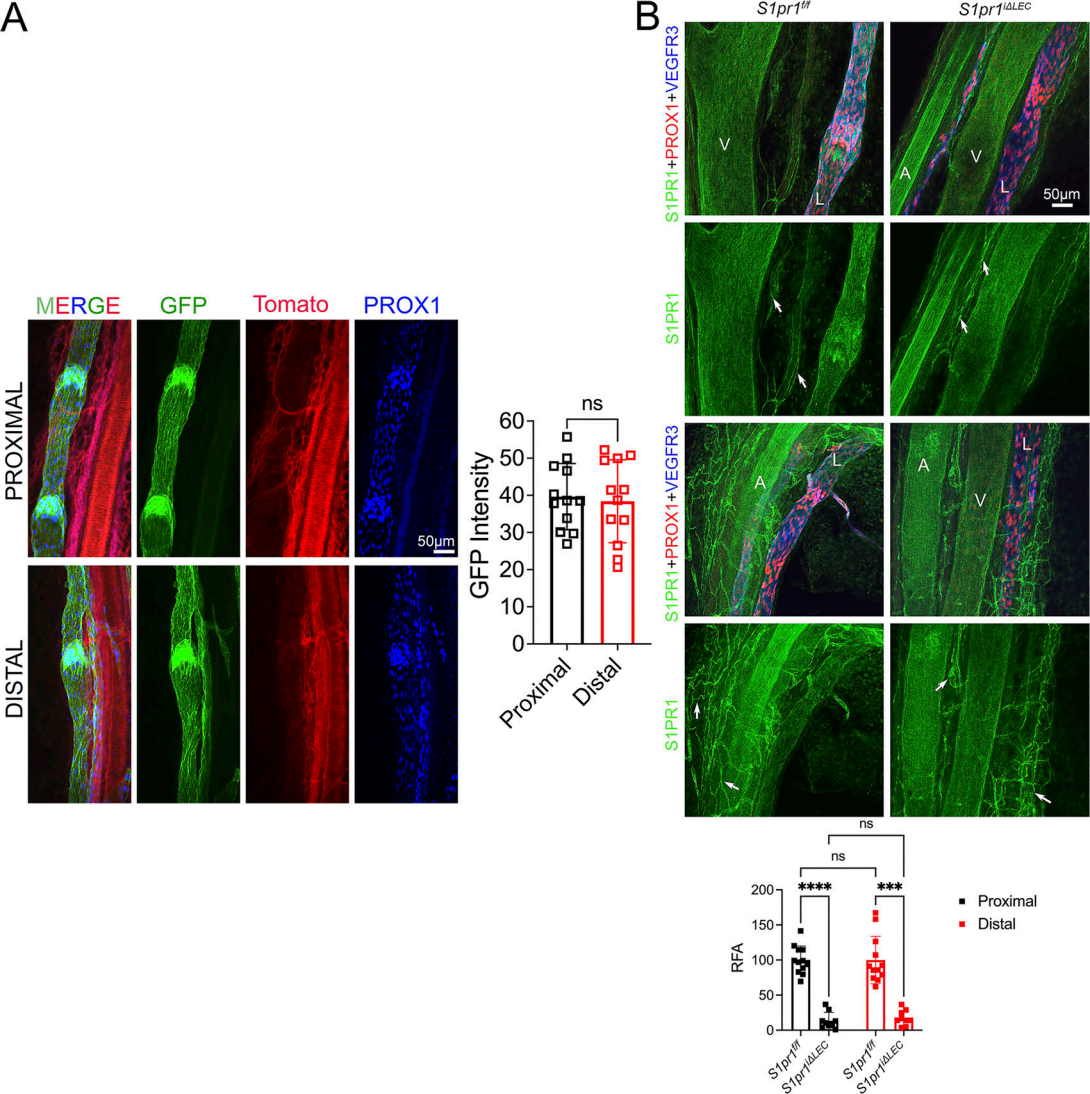
**Tg(Prox1-CreERT2) efficiently targets the entire mesenteric lymphatic vasculature and depletes S1PR1. (A)** The proximal and distal mesenteric vasculature of P10 Tg(Prox1-CreERT2);*R26*^*mT/mG*^ (TM@P1–7) pups were analyzed by IHC for PROX1 and autofluorescence of membrane-targeted GFP and membrane-targeted tdTomato. Downregulation of tdTomato and upregulation of GFP confirmed the efficient targeting of proximal and distal mesenteric lymphatic vessels by Tg(Prox1-CreERT2). GFP signal was semiquantitatively measured and plotted. **(B)** The proximal and distal mesenteric vasculature of P10 *S1pr1*^*f/f*^ (TM@P1–7) and *S1pr1*^*iΔLEC*^ (TM@P1–7) littermates were analyzed using the indicated antibodies. Semiquantitative measurement of the fluorescence signal confirmed that S1PR1 was specifically and efficiently deleted from the lymphatic vessels of *S1pr1*^*iΔLEC*^ pups. Arrows point to blood capillaries in which S1PR1 expression was comparable between the two genotypes. RFA, relative fluorescence activity. Scale bar is 100 μm. Statistics: (A) *n* = 4. Each dot represents the fluorescent intensity of a single vessel. Three proximal and three distal vessels from each mesentery were measured. **(B)***n* = 4 *S1pr1*^*f/f*^; *n* = 3 *S1pr1*^*iΔLEC*^ pups. Each dot represents the RFA of a single vessel. Three proximal and three distal vessels from each mesentery were measured. The graphs were plotted as mean ± SD. Unpaired *t* test (A) and two-way ANOVA (B) were performed for the statistical analysis. ***P < 0.001; ****P < 0.0001.

*Lyve1-Cre; S1pr1* ^*−/f*^ mice have mispatterned mesenteric blood and lymphatic vessels due to Cre activity in both cell types in this organ (Geng et al., 2020). Therefore, we used alternative approaches to investigate the role of S1PR1 in postnatal LV development. First, we used the S1PR1-GS mice to test if S1PR1 signaling is active in the mesenteric LVs. Analysis of the mesenteries of P10 S1PR1-GS pups revealed that S1PR1 signaling is active in the mesenteric collecting lymphatic vessels, although it is most potently expressed in LVs (Fig. 2 A, arrowheads). Next, to prevent mesenteric blood vascular defects, we used transgenic Prox1-CreERT2 (Tg[Prox1-CreERT2]) to delete *S1pr1* from LECs (Bazigou et al., 2011; Allende et al., 2003). First, we performed lineage tracing to estimate the gene deletion efficiency of Tg(Prox1-CreERT2). We generated Tg(Prox1-CreERT2); *R26*^*mT/mG*^ pups and fed them 1 μl of 20 mg/ml tamoxifen (TM) on postnatal day (P)1, 3 μl on P3, and so on until P7 (TM@P1–7). Subsequently, the pups were analyzed at P10. The *R26*^*mT/mG*^ reporter mice will express a membrane-targeted tdTomato reporter (mT) in the absence of Cre. The cDNA for mT will be deleted by TM-activated Cre recombinase and replaced by the cDNA for a membrane-targeted GFP reporter (mG). Thus, GFP expression is a readout of CreERT2-mediated gene deletion efficiency. We observed uniform GFP labelling of the mesenteric lymphatic vessels of Tg(Prox1-CreERT2); *R26*^*mT/mG*^ pups confirming the efficiency of Tg(Prox1-CreERT2) (Fig. S2 A).

Next, to delete *S1pr1*, we fed TM to Tg(Prox1-CreERT2);*S1pr1*^*f/f*^ (*S1pr1*^*iΔLEC*^) pups and their control littermates. We generated P10 *S1pr1*^*iΔLEC*^ (TM@P1–7) pups and confirmed the downregulation of S1PR1 in the mesenteric lymphatic vessels (Fig. S2 B).

**Figure S3.**
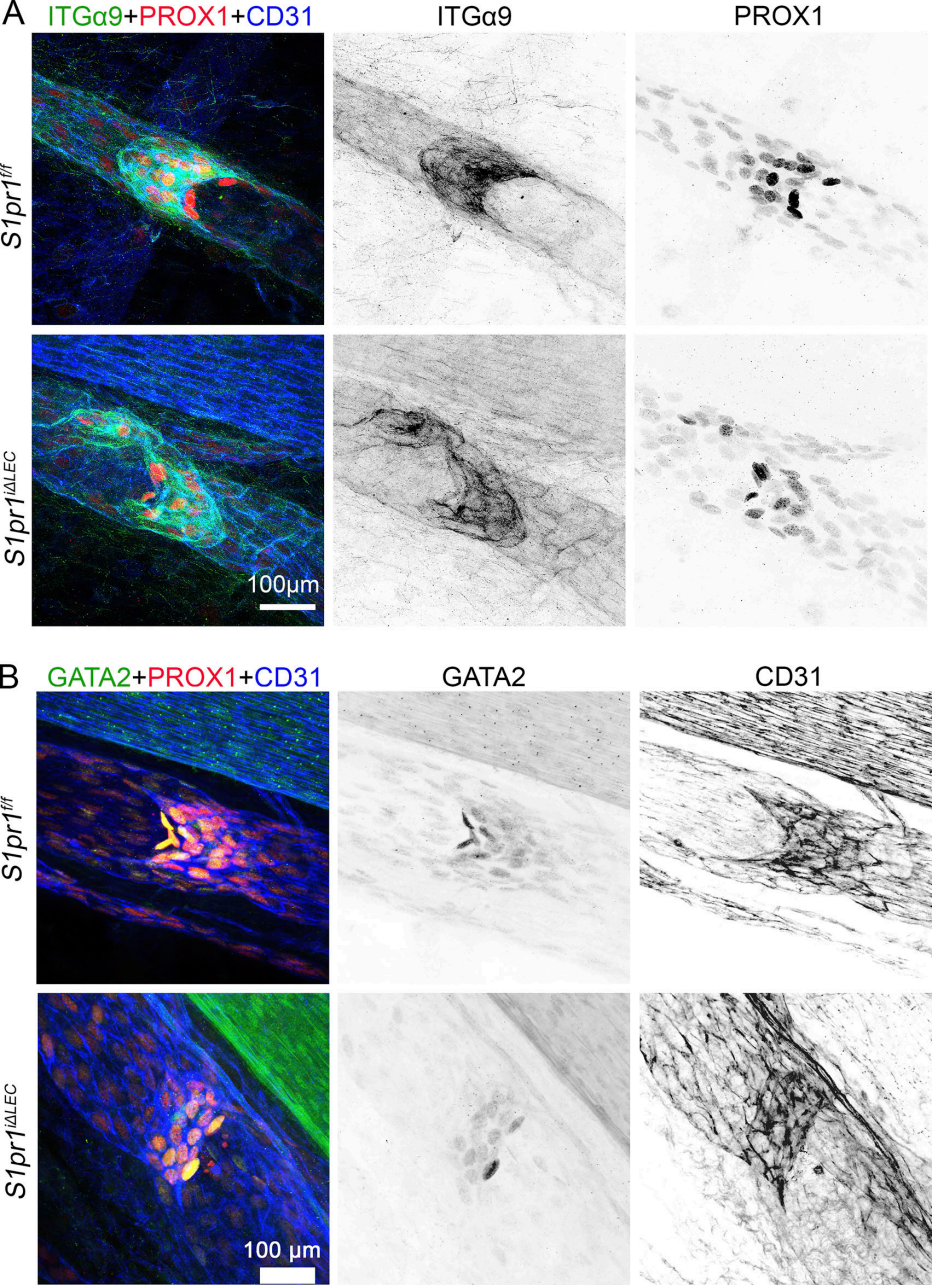
**Remaining LVs after efficient deletion of S1PR1 express valve markers normally.** The mesenteric vasculature of P10 *S1pr1*^*f/f*^ (TM@P1–7) and *S1pr1*^*iΔLEC*^ (TM@P1–7) littermates was analyzed using the indicated antibodies. **(A and B)** The expression of valve markers PROX1 (A and B), GATA2 (B), and integrin-α9 (A) appeared normal in the remaining LVs of mutants. Statistics: *n* = 3 pups per genotype.

A significant reduction in the number of mesenteric LVs was observed in *S1pr1*^*iΔLEC*^ pups (Fig. 2 B). Furthermore, LVs were more severely reduced in the mesenteric lymphatic vessels that drain the distal small intestine (ileum) when compared with the proximal small intestine (duodenum and jejunum). The valve markers PROX1, GATA2, and integrin-α9 were normally expressed in the remaining LVs (Fig. S3, A and B), although the expression of the gap junction molecule connexin-37 (CX37) appeared to be reduced (Fig. 2 C). Thus, S1PR1 is necessary for the postnatal development of mesenteric LVs.

**Figure 3.**
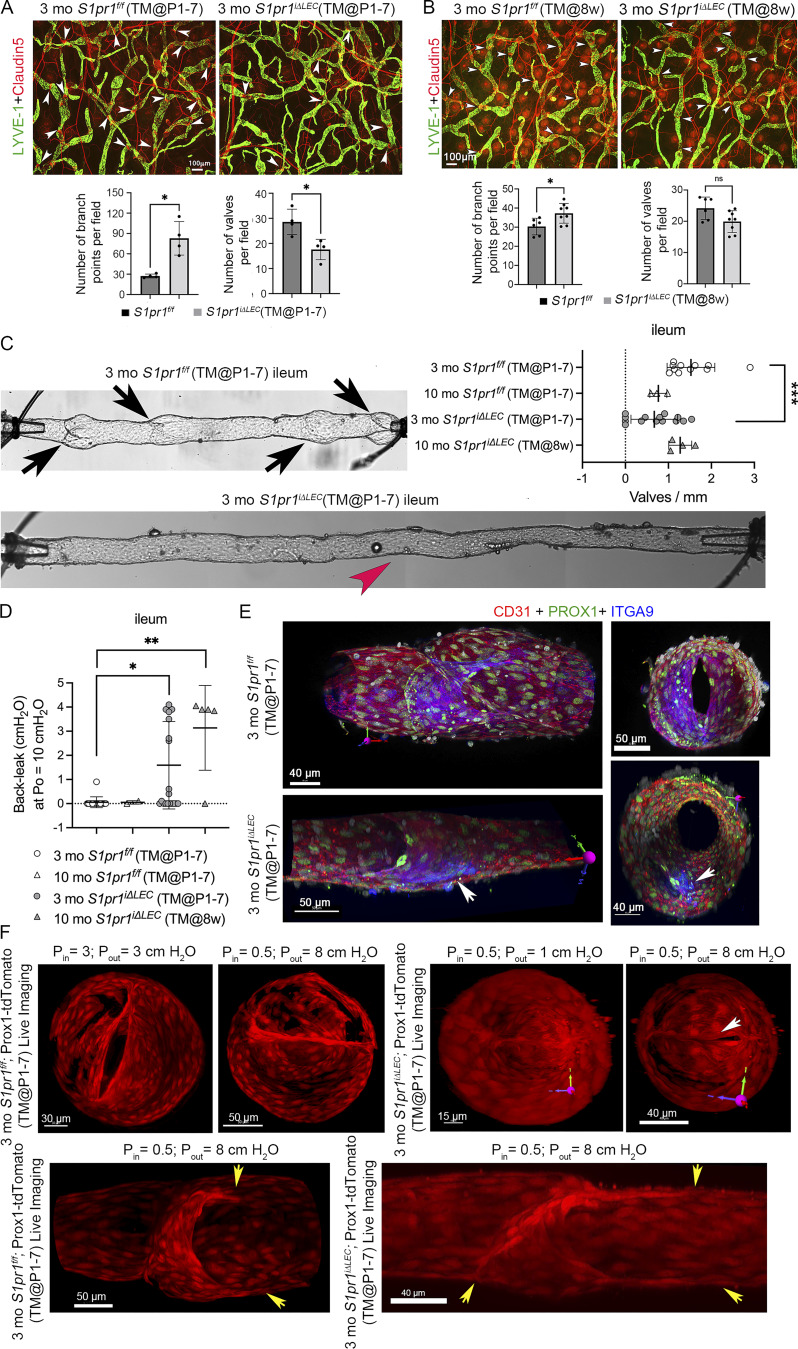
**LVs are reduced in the numbers and defective in *S1pr1*** ^***iΔLEC***^ **mice. (A and B)** 3-mo-old *S1pr1*^*iΔLEC*^ mice that were treated with TM either from P1–P7 (A) or at 8 wk of age (B) were studied. **(A)** Dermal lymphatic vessels in the ears of *S1pr1*^*iΔLEC*^ (TM@P1–P7) mice had more branches and fewer claudin-5^hi^ LVs (arrowheads) when compared with control littermates. **(B)***S1pr1*^*iΔLEC*^ (TM@8w) mice had elevated number of branch points but did not have any obvious reduction in LVs. **(C)** Representative stitched image of a terminal ileum-draining mesenteric lymphatic vessel of a control mouse with LVs (arrows) is shown. A corresponding stitched image of a lymphatic vessel from an *S1pr1*^*iΔLEC*^ (TM@P1–7) mouse lacking LVs is also shown (red arrowhead indicates where small nubs remain from a valve). The graph shows that the LV density is significantly reduced in *S1pr1*^*iΔLEC*^ (TM@P1–7) but not in *S1pr1*^*iΔLEC*^ (TM@8w) mice. **(D)** Ex vivo analysis of LVs in the ileum-draining lymphatic vessels. LVs of control, *S1pr1*^*iΔLEC*^ (TM@P1–7), and *S1pr1*^*iΔLEC*^ (TM@8w) mice were analyzed for back leak. The graph shows that the LVs in the terminal ileum-draining lymphatic vessels of mutant mice were significantly leaky irrespective of the time of gene deletion. **(E)** Following the back leak analysis, some leaky valves fixed for subsequent immunofluorescence to assess the specific LV structural component(s). IHC was performed on isolated vessel for the indicated markers, imaged by confocal microscopy, and 3D reconstructed. The 3D images were rotated to visualize the LVs on their side (left) or en face (right). LVs with two symmetrical leaflets were observed in control mice. However, in 1 of 6 *S1pr1*^*iΔLEC*^ (TM@P1–7) LVs imaged, only one partial leaflet (arrows, Prox1^Hi^-ITGA9^+^) was observed at the LV site, which resulted in complete back leak. **(F)** We performed live confocal imaging on 3 *S1pr1*^*iΔLEC*^ (TM@P1–7) LVs that exhibited various levels of back leak and two control LVs without back leak using the Prox1-tdTomato reporter under various levels of Pin and Pout. Both control and mutant LVs remained open when Pin and Pout were equal and closed when Pout was slightly elevated. The control LV remained closed when Pout was increased to 8 cm H_2_O. In contrast, a gap remained in a mutant LV (arrow), resulting in back leak. Symmetrically located commissures that extend in the downstream direction can be observed in the same control LV visualized from the side (yellow arrows). In contrast, the *S1pr1*^*iΔLEC*^ (TM@P1–7) LV had asymmetrical commissures that extended in both upstream and downstream directions (yellow arrows). Statistics: (A and B) Each dot represents an individual mouse. The graphs were plotted as mean ± SD. Mann–Whitney test and unpaired *t* test were performed for the statistical analysis. *P < 0.05. **(C)** LV density was measured in ileum-draining lymphatic vessels harvested from *n* = 10 3-mo *S1pr1*^*f/f*^ (TM@P1–P7), *n* = 5 10-mo *S1pr1*^*f/f*^ (TM@P1–P7), *n* = 11 3-mo *S1pr1*^*iΔLEC*^ (TM@P1–P7), and *n* = 3 10-mo *S1pr1*^*iΔLEC*^ (TM@8w) mice. One-way ANOVA with Tukey’s post hoc test was performed to determine significance. ***P < 0.001. **(D)** Each dot represents an individual LV harvested from *n* = 10 3-mo *S1pr1*^*f/f*^ (TM@P1–P7), *n* = 2 10-mo *S1pr1*^*f/f*^ (TM@P1–P7), *n* = 11 3-mo *S1pr1*^*iΔLEC*^ (TM@P1–P7), and *n* = 3 10-mo *S1pr1*^*iΔLEC*^ (TM@8w) mice. A nonparametric Kruskal–Wallis test with Dunn’s post hoc test was performed to determine significance. *P < 0.05; **P < 0.01. **(E and F)** Live imaging followed by fixation and whole-mount IHC was performed using *n* = 2 LVs from 3-mo *S1pr1*^*f/f*^;Prox1-tdTomato (TM@P1–P7) and *n* = 3 LVs from 3-mo *S1pr1*^*iΔLEC*^;Prox1-tdTomato (TM@P1–P7) mice. Additionally, *n* = 3 LVs that were not imaged live from 3-mo *S1pr1*^*iΔLEC*^ (TM@P1–P7) mice were directly fixed and imaged by whole-mount IHC.

To determine whether LVs are defective in other lymphatic vascular beds, we analyzed the ears of 3-mo-old *S1pr1*^*iΔLEC*^ (TM@P1–7) mice with claudin-5, which is a reliable marker for both endothelial cell tight junctions and LVs (Frye et al., 2020). We determined that the dermal lymphatic vessels had a significantly higher number of branch points but fewer claudin-5^+^ LVs when compared with their control littermates (Fig. 3 A). Thus, S1PR1 is necessary for the development of dermal LVs. To determine if S1PR1 is necessary for the maintenance of already formed LVs, we administered TM by gavage to 8-wk-old mice for 3 consecutive days. The *S1pr1*^*iΔLEC*^ (TM@8w) mice and their control littermates were analyzed 4 wk later. No obvious increase in lymphatic vessel branch point density or reduction in LV numbers was observed in the ear lymphatic vessels (Fig. 3 B). Therefore, S1PR1 is not necessary to maintain the number of already formed dermal LVs.

We analyzed the mesenteric lymphatic vessels of 3-mo-old control and *S1pr1*^*iΔLEC*^ (TM@P1–7) mice. The ileum-draining lymphatic vessels from control mice had 1–3 LVs per mm of vessel (Fig. 3 C). In contrast, lymphatic vessels from *S1pr1*^*iΔLEC*^ (TM@P1–7) mice had fewer LVs, and some were devoid of LVs (Fig. 3 C, red arrowhead). However, no obvious reduction in LV numbers was observed in 10-mo-old *S1pr1*^*iΔLEC*^ (TM@8w), indicating that S1PR1 is necessary for the formation but not for maintaining the number of LVs.

**Figure S4.**
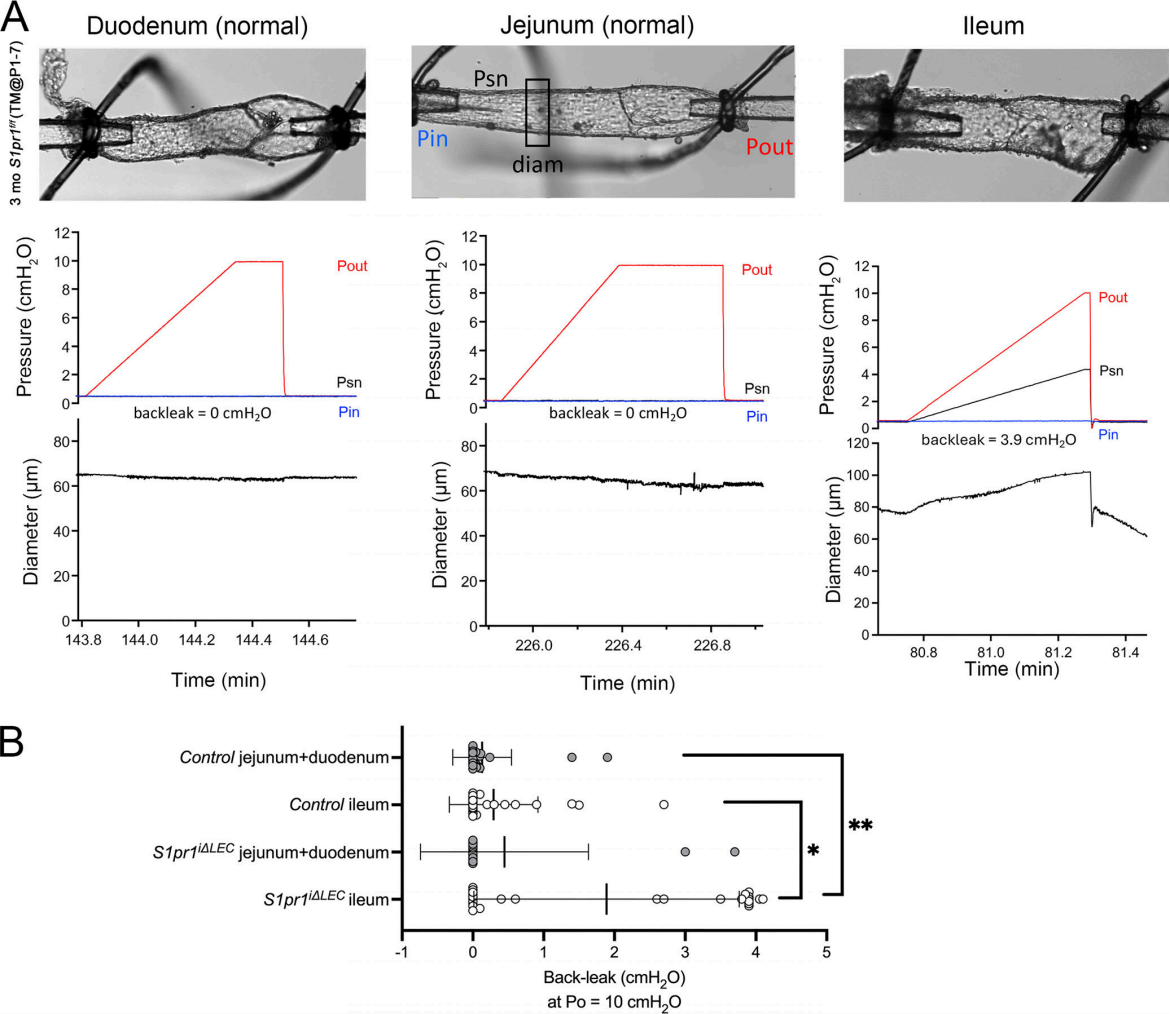
**Most LVs located in the proximal mesenteric lymphatic vessels of *S1pr1*** ^***iΔLEC***^ **mice were normal. (A)** Mesenteric lymphatic vessels from the sections corresponding to the duodenum, jejunum, and ileum were dissected from 3-mo-old *S1pr1*^*iΔLEC*^ (TM@P1–7) mice, cleaned, and cannulated (top row). Subsequently, LV function test was performed. While gradually increasing the Pout, the pressure (Psn) and vessel diameter were measured behind the LVs. Duodenal and jejunal LVs did not have any pressure back leak (second row). Consequently, those vessels did not expand in diameter as Pout was raised (bottom row). In contrast, an LV from the ileum exhibited back leak, as indicated by increasing Psn and diameter with increasing Pout. **(B)** Quantification of back leak of LVs harvested from the duodenum and jejunum versus ileum of control and *S1pr1*^*iΔLEC*^ mice. Statistics: (B) *n* = 31 control (duodenum and jejunum), *n* = 28 control (ileum), *n* = 15 *S1pr1*^*iΔLEC*^ (duodenum and jejunum), and *n* = 26 *S1pr1*^*iΔLEC*^ (ileum) LVs. LVs from 3-mo-old *S1pr1*^*iΔLEC*^ (TM@P1–P7) and 10-mo-old *S1pr1*^*iΔLEC*^ (TM@8w) were combined as *S1pr1*^*iΔLEC*^ for this analysis. *P < 0.05; **P < 0.01.

Next, mesenteric lymphatic vessels from various regions of the gut were isolated and cannulated to test LV function as described previously (Lapinski et al., 2017). LVs in the duodenum-, jejunum-, or ileum-draining lymphatic vessels of 3- and 10-mo-old control mice closed upon elevation of outflow pressure (Pout) and completely prevented back leak (Fig. 3 D and Fig. S4, A and B). Most LVs in the duodenum- and jejunum-draining lymphatic vessels of *S1pr1*^*iΔLEC*^ (TM@P1–7) mice were also normal (Fig. S4, A and B). In contrast, significant numbers of LVs in the ileum-draining lymphatic vessels of *S1pr1*^*iΔLEC*^ (TM@P1–7) mice were leaky (Fig. 3 D). We also analyzed the ileal LVs of 10-mo-old *S1pr1*^*iΔLEC*^ mice (TM@8w). Although these mice had normal numbers of LVs, they were significantly leaky (Fig. 3 D). Thus, S1PR1 is necessary to maintain the normal functioning of ileal LVs.

**Figure S5.**
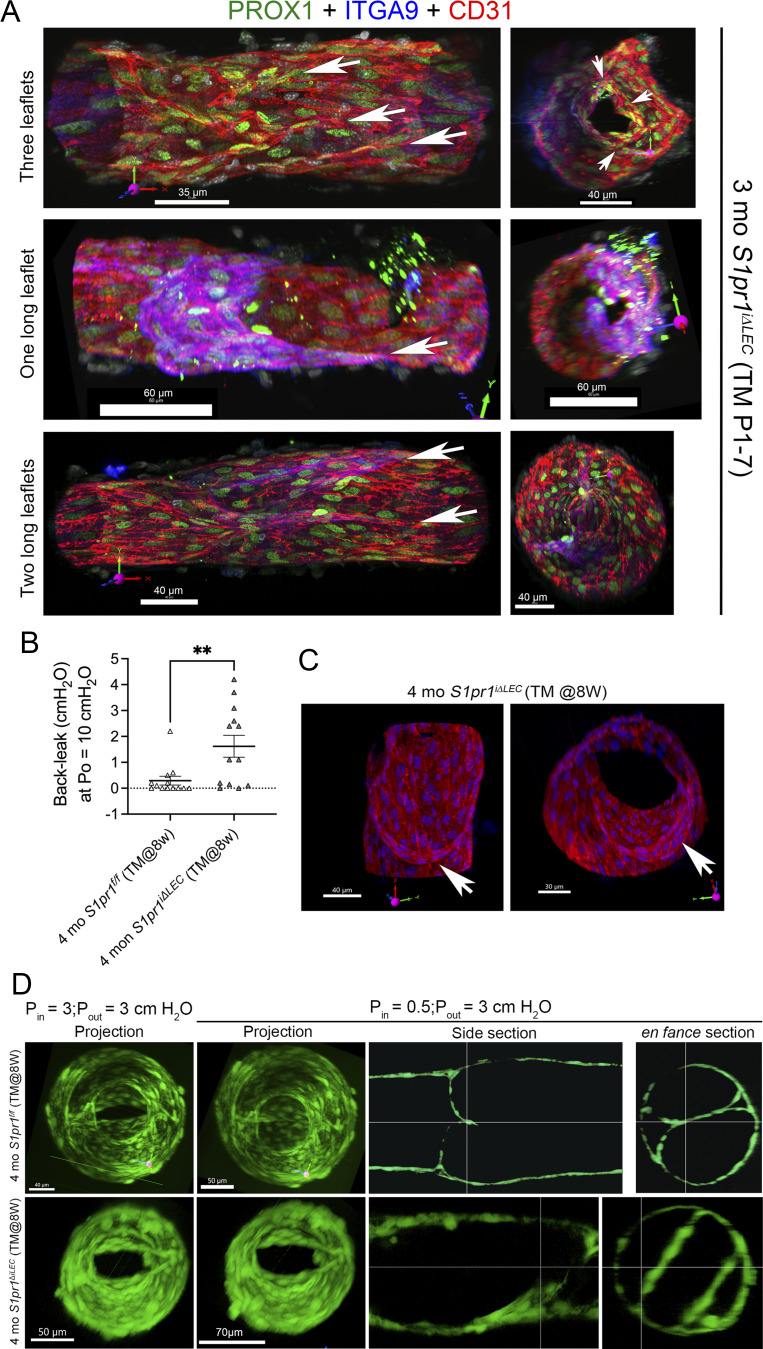
**Heterogeneous LV defect in *S1pr1*** ^***iΔLEC***^ **mice. (A)** Lymphatic vessels with leaky LVs from 3-mo-old *S1pr1*^*iΔLEC*^ (TM@P1–7) mice were fixed, and whole-mount IHC was performed for the indicated markers. Confocal imaging and 3D reconstruction were performed to identify the structural defects in the LVs. One LV appeared to have three leaflets (arrows). One or both leaflets were abnormally elongated at their insertion points in two other LVs, resulting in abnormal en face LV structure (right). **(B)** Back leak test revealed significant leakage in the ileal LVs of 4-mo-old *S1pr1*^*iΔLEC*^ (TM@8w). **(C)** A leaky LV from a 4-mo-old *S1pr1*^*iΔLEC*^ (TM@8w) mouse was fixed, and whole-mount IHC was performed for PROX1 (blue) and CD31 (red). Confocal imaging and 3D reconstruction revealed a single leaflet (arrow). **(D)** Lymphatic vessels were incubated with CellTracker Green (CMFDA) stain and imaged live under various Pins and Pouts. The confocal images were 3D reconstructed or analyzed at various planes. Control LV was open when Pin = Pout and closed when Pout > Pin. Tight overlap between leaflets can be observed in digital sections. In contrast, a leaky LV from a 4-mo-old *S1pr1*^*iΔLEC*^ (TM@8w) mouse remained open when Pout > Pin. Digital sections revealed short leaflets that did not overlap. Statistics: (A) Live imaging followed by fixation and whole-mount IHC was performed using *n* = 2 LVs from 3-mo-old *S1pr1*^*f/f*^;Prox1-tdTomato (TM@P1–P7) and *n* = 3 LVs from 3-mo-old *S1pr1*^*iΔLEC*^;Prox1-tdTomato (TM@P1–P7) mice. Additionally, *n* = 3 LVs that were not imaged live from 3-mo-old *S1pr1*^*iΔLEC*^ (TM@P1–P7) mice were directly fixed and imaged by whole-mount IHC. (B) *n* = 13 LVs from *n* = 8 4-mo-old *S1pr1*^*f/f*^ (TM@8w) mice and *n* = 13 LVs from *n* = 5 4-mo-old *S1pr1*^*iΔLEC*^ (TM@8w) mice were analyzed for back leak. **P < 0.01. **(C and D)** Following back leak test (B), *n* = 5 LVs from 4-mo-old *S1pr1*^*f/f*^ (TM@8w) mice and *n* = 8 leaky LVs from 4-mo-old *S1pr1*^*iΔLEC*^ (TM@8w) mice were analyzed by live imaging, followed by fixation and whole-mount IHC. In addition to those 8, the single leaflet valve from the *S1pr1*^*iΔLEC*^ (TM@8w) mouse was assessed only by fixation and whole-mount IHC (C). **(D)** Under live imaging at high pressure, three control valves from 4-mo-old *S1pr1*^*f/f*^ (TM@8w) mice were completely normal. A representative control valve is shown in D. One control valve had a small gap at the commissure at the high pressure. The remaining valve had 1 dysfunctional commissure where the annulus failed to meet and asymmetrical leaflet insertions into the sinus, which prevented it from closing in response to adverse pressure. Of the *S1pr1*^*iΔLEC*^ (TM@8w) LVs at high pressures, four LVs failed to close while three were partly closed and one tightly closed. From the four leaky LVs that failed to close, one had short leaflets that did not reach the midpoint of the lumen (shown in D). In two of the four leaky LVs and two of the three partly leaky LVs, there was asymmetry in the leaflet’s upstream insertion site.

To determine the structural defects in leaky LVs, we performed live imaging on LVs immediately after conducting valve tests. We harvested LVs from Prox1-tdTomato and *S1pr1*^*iΔLEC*^; Prox1-tdTomato (TM@P1–7) mice and imaged them live at various levels of inflow pressure (Pin) and Pout. Control LVs closed at low Pout and remained tightly closed at high Pout (Fig. 3 D). In contrast, a mutant LV closed at low Pout but developed gaps at high Pout (Fig. 3 D, arrow). We also performed IHC on isolated vessels after fixation and imaged them by confocal microscopy followed by 3D reconstruction of the LVs. While control valves had two symmetrical leaflets, a leaky valve from an *S1pr1*^*iΔLEC*^ (TM@P1–7) mouse had only one leaflet (Fig. 3 D, arrows). Another leaky valve appeared to have three leaflets (Fig. S5 A). One or both leaflets were abnormally elongated at their insertion points in the wall in two other leaky LVs (Fig. S5 A). We observed similar structural defects in the leaky LVs of 4-mo-old *S1pr1*^*iΔLEC*^ (TM@8w) as well (Fig. S5, B–D). These results indicated that S1PR1 is necessary to maintain the symmetry of LV leaflets and the formation of proper commissures. The significant heterogeneity in LV defects is consistent with the defects that were observed in LVVs (Fig. 1 B).

In summary, S1PR1 is necessary for the postnatal development of LVs and prevent back leak under adverse pressure gradient. Intriguingly, LVs in the ileum-draining lymphatic vessels are more sensitive to the loss of S1PR1 when compared with more proximally located LVs, thus highlighting a previously unknown heterogeneity within mesenteric lymphatic vessels.

### Lymphatic drainage is defective, and TLOs are present in the mesenteries of *S1pr1*^*iΔLEC*^ mice

**Figure 4.**
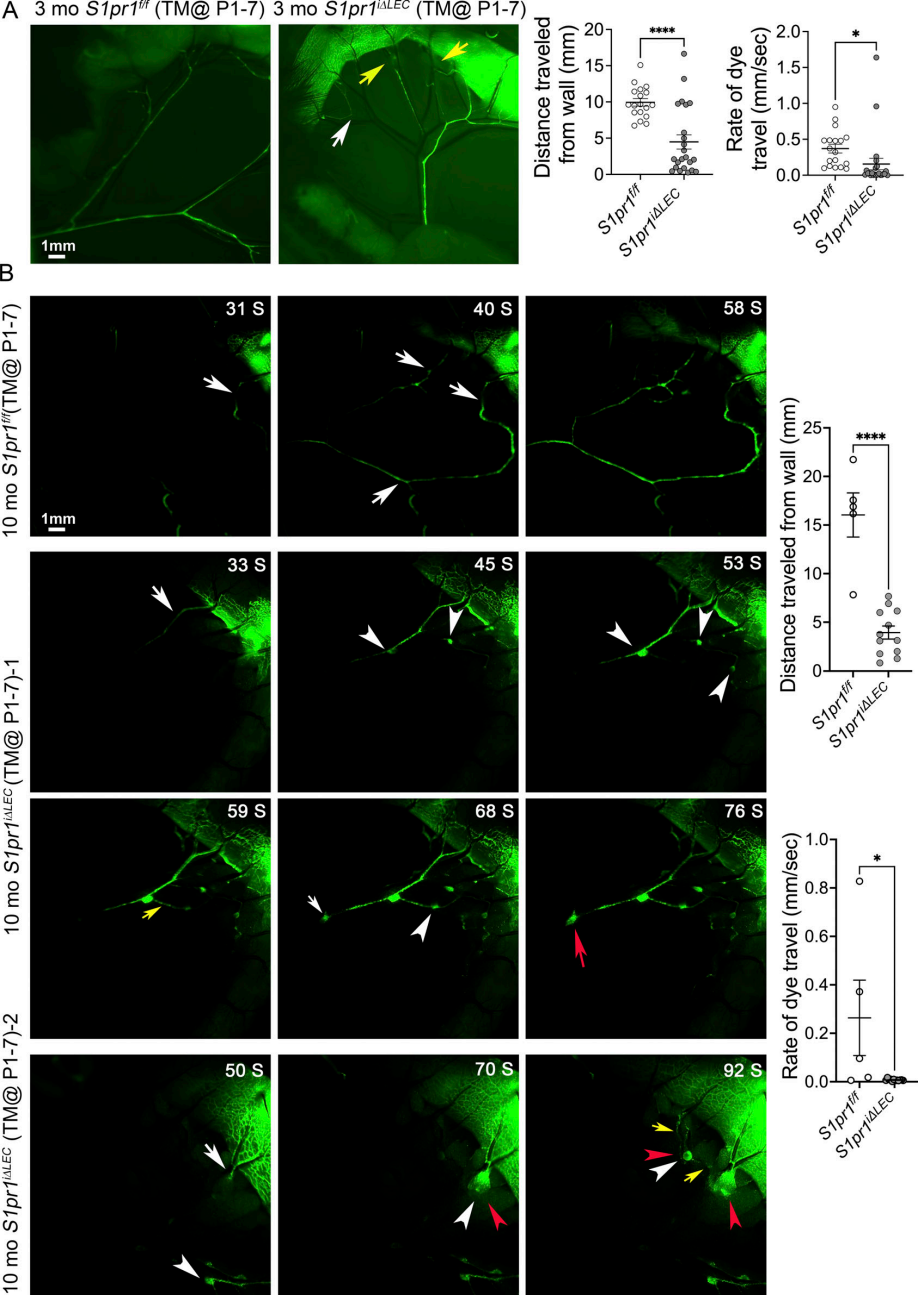
**Lymphatic drainage is defective and obstructed by nodules in *S1pr1*** ^***iΔLEC***^ **mice. (A and B)** FITC-conjugated dextran (2,000 kD) was injected into the muscle layer of the ileum and/or the Peyer’s patches of anesthetized 3-mo-old (A) or 10-mo-old (B) *S1pr1*^*f/f*^ and *S1pr1*^*iΔLEC*^ mice that were treated with TM from P1–7. The flow of fluorescent dye through the mesenteric lymphatic vessels was visualized by live imaging. **(A)** The dye rapidly drains through the lymphatic vessels in control mice. In contrast, the dye abruptly stopped (white arrow) or appeared to bypass certain locations (yellow arrows) in mutant mice. The graphs show that the distance travelled by the dye and the rate at which the dye travelled were significantly reduced in *S1pr1*^*iΔLEC*^ mice. **(B)** Time in seconds after injection is indicated on the top right corner of the panels. The dye rapidly drains through the lymphatic vessels in control mice (white arrows). In contrast, the dye accumulated in nodules that were connected to the lymphatic vessels in the mutant mice (white arrowheads). Retrograde flow was also observed between the nodules (yellow arrows). The nodules were observed both in pre-collecting vessels (red arrowheads) and in collecting lymphatic vessels (red arrow). The videos were analyzed to quantify the distance travelled by the dye and the rate of travel. The graphs show that these parameters were significantly reduced in *S1pr1*^*iΔLEC*^ mice. Statistics: Images are representative of (A) *n* = 3 *S1pr1*^*f/f*^ and *n* = 4 *S1pr1*^*iΔLEC*^ mice; (B) *n* = 4 *S1pr1*^*f/f*^ and *n* = 5 *S1pr1*^*iΔLEC*^ mice. Some samples were analyzed by injection at multiple sites. Each dot in the graph indicates an individual injection. Graphs were plotted as mean ± SEM. Unpaired *t* tests were performed for the statistical analyses. *P < 0.05; ****P < 0.0001.

As the LVs were defective in the terminal ileum of *S1pr1*^*iΔLEC*^ mice, we wanted to determine if lymphatic drainage is defective in these mice. We injected FITC-conjugated dextran (molecular weight = 2000 kD) into the muscle layer of the gut wall and/or the Peyer’s patches of anesthetized 3-mo-old mice and performed live imaging to visualize the flow of fluorescent dye. In control mice, the dye was drained by the mesenteric-collecting lymphatic vessels quickly and in a unidirectional manner (Fig. 4 A). In *S1pr1*^*iΔLEC*^ mice dye drained normally in some vessels (Fig. 4 A). However, dye appeared to abruptly stop in certain vessels (Fig. 4 A, white arrow) and bypass certain locations in other vessels (Fig. 4 A, yellow arrows). Overall, the distance travelled by the dye and the rate of dye flow were significantly reduced in *S1pr1*^*iΔLEC*^ mice.

We repeated the fluorescence lymphangiography in 10-mo-old mice. In control mice, the dye drained normally as before (Fig. 4 B, arrows and Video 1). In contrast, in *S1pr1*^*iΔLEC*^ mice dye drained into numerous nodules that appeared to slow down the flow (Fig. 4 B, arrowheads and Videos 2 and 3). Additionally, dye often appeared to flow in the retrograde direction from nodule to nodule (Fig. 4 B, yellow arrows). However, no obvious leakage of dye was observed from the lymphatic vessels or nodules. The nodules were observed both in pre-collecting vessels (Fig. 4 B, red arrowheads) and in collecting lymphatic vessels (Fig. 4 B, red arrow).

**Video 1.** **Live imaging of fluorescent dye flow along the ileal lymphatic vessels of a 1-year-old *S1pr1*** ^***f/f***^ **mouse.**

**Video 2.** **Live imaging of fluorescent dye flow along the ileal lymphatic vessels of a 1-year-old *S1pr1*** ^***iΔLEC***^ **mouse (sample 1).**

**Video 3.** **Live imaging of fluorescent dye flow along the ileal lymphatic vessels of a 1-year-old *S1pr1*** ^***iΔLEC***^ **mouse (sample 2).**

**Figure 5.**
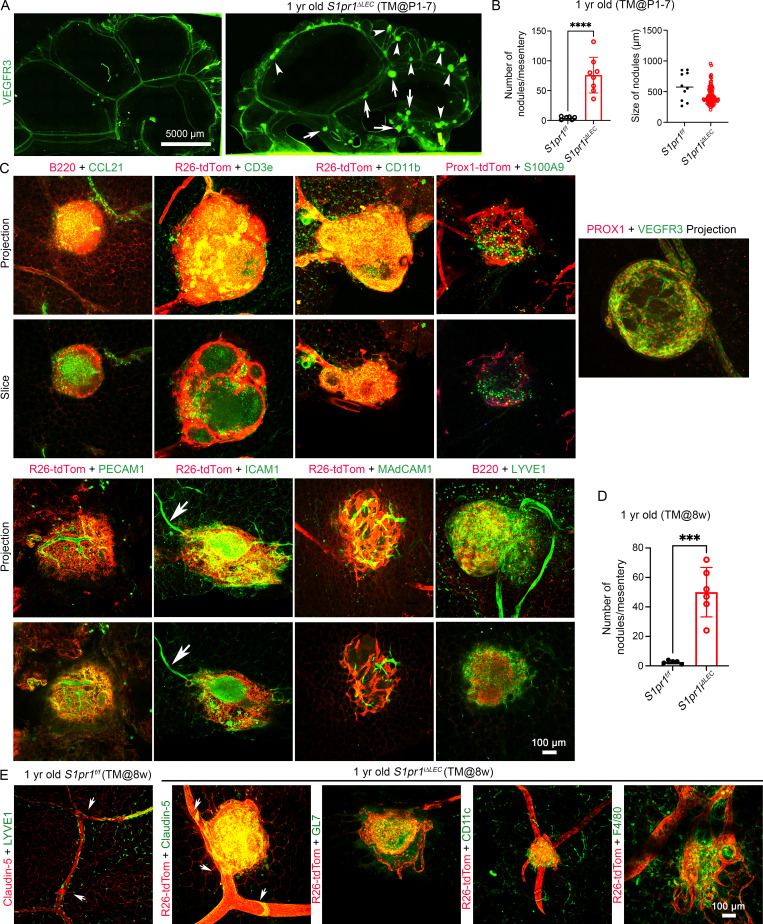
**TLOs are present in the terminal ileum of *S1pr1*** ^***iΔLEC***^ **mice, and they are connected to the lymphatic vessels. (A)** Mesenteric tissue along the ileum was harvested and analyzed using the lymphatic vessel marker VEGFR3. Stitched images revealed a large number of nodules in *S1pr1*^*iΔLEC*^ (TM@P1–7) mice, but not in control littermates. The nodules were observed both in pre-collecting vessels (arrowheads) and in collecting lymphatic vessels (arrows). **(B)** The number and size of LYVE1^+^B220^+^ nodules were measured and quantified. **(C)** Mesenteries of 8–12-mo-old control mice and *S1pr1*^*iΔLEC*^ littermates that were treated with TM from P1–7 were analyzed using markers for the immune cells, stromal cells, and endothelial cells. Some *S1pr1*^*iΔLEC*^ mice had *R26*^*+/tdTomato*^ reporter, the expression of which was permanently induced in the lymphatic vessels by TM-activated CreERT2. A few other mice had the Prox1-tdTomato reporter. Lymphatic vessels were labelled by VEGFR3, PROX1, LYVE1, and tdTomato. LYVE1 was also expressed in a subset of macrophages. B220, CD3e, CD11b, and S100A9 are markers of B cells, T cells, myeloid lineage cells, and neutrophils, respectively. CCL21 is a marker for lymphatic vessels and the FRCs within TLOs. PECAM1 labels all endothelial cells, but its expression is stronger in blood endothelial cells when compared with LECs. ICAM1 is expressed in HEVs, inflamed blood endothelial cells, and a variety of immune cells. MAdCAM1 is a marker of HEVs. **(D)** TLOs in the terminal ileum of 1-year-old *S1pr1*^*f/f*^ and *S1pr1*^*iΔLEC*^ (TM@8W) mice were counted and plotted. **(E)** Mesenteric lymphatic vessels of *S1pr1*^*f/f*^ mice had clear claudin-5^+^ LVs (arrows) and weak LYVE1 expression. LVs were also observed in *S1pr1*^*iΔLEC*^ (TM@8W) mice (arrows), although those that were close to the TLOs appeared defective. The TLOs had GL7^+^ germinal center B cells, CD11c^+^ dendritic cells, and F4/80^+^ macrophages. Statistics: (A) *n* = 3 *S1pr1*^*f/f*^ and *n* = 3 *S1pr1*^*iΔLEC*^ (TM@P1–7) mice; (B) *n* = 6 *S1pr1*^*f/f*^ and *n* = 8 *S1pr1*^*iΔLEC*^ mice. The size of the nodules was quantified, and each dot represents a nodule on the graph; (C and E) representative images from three to five mice/genotype/marker; (D) *n* = 6 *S1pr1*^*f/f*^ and *n* = 7 *S1pr1*^*iΔLEC*^ (TM@8W) mice. Graphs were plotted as mean ± SD. Welch’s *t* test (B [number of TLOs] and D) and Mann–Whitney test (B [TLO size]) were performed for the statistical analysis. ***P < 0.001; ****P < 0.0001.

Recently, TLOs were found to form in the terminal ileum of *Tnf*^*+/ΔARE*^ mice, a model for ileitis (Rehal and von der Weid, 2017; Czepielewski et al., 2021). The nodules in the mesentery of *S1pr1*^*iΔLEC*^ mice were reminiscent of TLOs of *Tnf*^*+/ΔARE*^ mice (Rehal and von der Weid, 2017; Czepielewski et al., 2021). Therefore, we characterized the mesenteric tissue by IHC with markers of immune, stromal, and vascular cells. IHC for the LEC marker VEGFR3 revealed that the nodules were primarily located in the terminal ileum of *S1pr1*^*iΔLEC*^ mice, as in *Tnf*^*+/ΔARE*^ mice (Fig. 5 A). This analysis further confirmed that the nodules were observed both in pre-collecting vessels and collecting vessels. On average, 60–70 nodules that measured ∼300 μm in diameter were observed in *S1pr1*^*iΔLEC*^ mice (Fig. 5 B). Some of the *S1pr1*^*iΔLEC*^ mice had *R26*^*+/tdTomato*^ reporter to permanently label PROX1^+^ lymphatic vessels at the time of TM injection. The *R26*^*+/tdTomato*^ allele has a LoxP-transcriptional stop signal-LoxP (LSL) cassette that is located downstream of the constitutively active *R26* regulatory element and upstream of the tdTomato fluorescent reporter. The LSL cassette prevents the expression of tdTomato. TM-activated Cre recombinase will recognize the LoxP sites and permanently delete the LSL cassette, resulting in the constitutive expression of tdTomato in Cre^+^ cells and their descendants. Expression of tdTomato, LYVE1, VEGFR3, and PROX1 revealed that the lymphatic vessels were wrapped around the nodules (Fig. 5 C). Furthermore, the *R26*^*+/tdTomato*^ lineage tracer revealed that these lymphatic vessels had originated from lymphatic vessels that existed at least as early as P1–7, the time of TM administration.

The nodules contained B220^+^ B cells, CD3e^+^ T cells, CD11b^+^ leukocytes, S100A9^+^ neutrophils, CCL21^+^ LECs, and CCL21^+^ FRCs that were located at the core of the nodules (Fig. 5 C). IHC for the pan-endothelial marker CD31/PECAM1 revealed the presence of tdTomato^−^ blood vessels within TLOs (Fig. 5 C). Lymphocytes enter the LNs from the blood circulation via HEVs (Drayton et al., 2006); some of the tdTomato^−^ blood vessels expressed the HEV marker MAdCAM1 (Fig. 5 C). Leukocytes extravasate from blood vessels through the interaction of CD11b with adhesion molecules such as ICAM1 that are expressed on inflamed endothelial cells (Ley et al., 2007) and HEVs (Vella et al., 2023). ICAM1 is also expressed in LTo cells and some leukocytes (Onder et al., 2017). ICAM1 was identified in blood vessels both within and outside the nodules of *S1pr1*^*iΔLEC*^ mice (Fig. 5 C, arrow). On the other hand, ICAM1 expression was patchy in the tdTomato^+^ lymphatic vessels. Based on the expression pattern of various markers, we concluded that the nodules in the terminal ileum of *S1pr1*^*iΔLEC*^ mice were TLOs that contain lymphatic vessels, inflamed blood vessels, HEVs, B cells, T cells, myeloid cells, and FRCs.

Finally, we harvested the mesenteries of 1-year-old *S1pr1*^*iΔLEC*^ mice that were exposed to TM at 8 wk of age and determined that they too had TLOs with CD11c^+^ DCs, GL7^+^ germinal center B cells, and F4/80^+^ macrophages in the terminal ileum (Fig. 5, D and E). Thus, S1PR1 is constantly required to prevent TLO formation in the terminal ileum and the appearance of TLOs correlates with the presence of defective LVs.

### LV development and TLO formation are regulated by cell-autonomous S1PR1 signaling in LECs

S1P is the ligand for S1PR1, and it is generated by a complex metabolic pathway consisting of numerous intermediates and regulatory enzymes (Maceyka and Spiegel, 2014). The final step of S1P synthesis is mediated by sphingosine kinases 1 and 2 (SPHK1/2), which convert sphingosine to S1P. S1P is secreted from hematopoietic cells by the transporter MFSD2B and from endothelial cells by the transporter SPNS2 (Cartier and Hla, 2019). Deletion of *Sphk1/2* or *Spns2* from LECs significantly downregulates S1P levels in lymph (Pham et al., 2010; Mendoza et al., 2012). Thus, LECs are the primary source of S1P in lymph.

**Figure 6.**
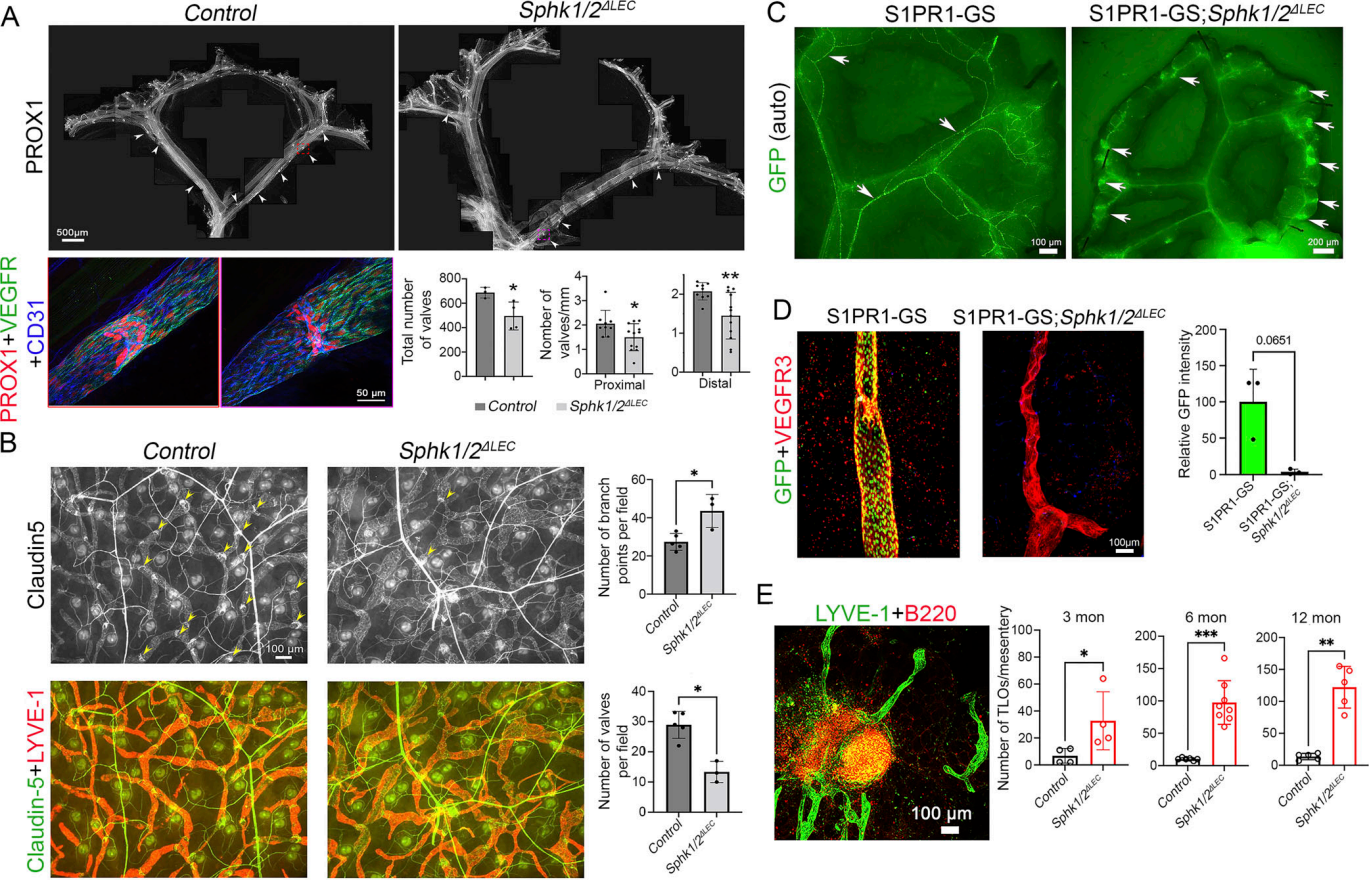
**Autocrine or paracrine S1PR1 signaling regulates LV development and TLO formation. (A and B)** Mesenteric (A) and dermal (B) lymphatic vessels of *Sphk1/2*^*ΔLEC*^ mice, in which S1P synthesis in LECs was ablated, were analyzed. **(A)** Stitched images of mesenteric lymphatic vessels revealed fewer LVs in P10 *Sphk1/2*^*ΔLEC*^ pups. The remaining LVs appeared immature (bottom row). Representative valves are within dotted boxes, and their enlarged images are shown below. **(B)** Dermal lymphatic vessels of 3-mo-old *Sphk1/2*^*ΔLEC*^ mice had fewer LVs (yellow arrows) and more branches per field. **(C)** The mesenteries of 1-year-old S1PR1-GS and S1PR1-GS;*Sphk1/2*^*ΔLEC*^ mice were analyzed. GFP autofluorescence was observed in the lymphatic vessels of S1PR1-GS mice (arrows). Weaker GFP expression was observed in blood vessels. In contrast, lymphatic vessels could not be identified based on GFP autofluorescence in S1PR1-GS;*Sphk1/2*^*ΔLEC*^ mice. However, GFP expression was observed in blood vessels and in nodule-like structures (arrows). **(D)** IF for GFP and VEGFR3 revealed a dramatic downregulation of GFP expression in the lymphatic vessels of S1PR1-GS;*Sphk1/2*^*ΔLEC*^ mice. **(E)** IF for LYVE1 and B220 revealed significant number of TLOs in 3-, 6- and 12-mo-old *Sphk1/2*^*ΔLEC*^ mice. The picture shows a representative TLO from a 12-mo-old *Sphk1/2*^*ΔLEC*^ mouse. Statistics: (A) *n* = 3 control and *n* = 4 *Sphk1/2*^*ΔLEC*^ pups. Total number of LVs in the entire mesentery were counted. Each dot in the graph represents an individual animal. Three proximal vessels and three distal vessels from each mesentery were analyzed to quantify the valve density. Each dot in the graph represents a vessel; (B) *n* = 4 controls and *n* = 3 *Sphk1/2*^*ΔLEC*^ mice. (C) *n* = 7 S1PR1-GS and *n* = 8 S1PR1-GS;*Sphk1/2*^*ΔLEC*^ mice; (D) *n* = 3 6-mo-old S1PR1-GS and *n* = 3 S1PR1-GS;*Sphk1/2*^*ΔLEC*^ littermates; (E) 3-mo-old mice: *n* = 4 control and *n* = 4 *Sphk1/2*^*ΔLEC*^; 6-mo-old mice: *n* = 6 control and *n* = 8 *Sphk1/2*^*ΔLEC*^; and 1-year-old mice: *n* = 5 control and *n* = 5 *Sphk1/2*^*ΔLEC*^. Graphs were plotted as mean ± SD. Unpaired *t* tests (A), Welch’s *t* tests (D), and Mann–Whitney tests (B and E) were performed for the statistical analysis. *P < 0.05; **P < 0.01; ***P < 0.001.

We generated P10 *Lyve1-Cre;Sphk1*^*−/f*^*;Sphk2*^*−/−*^ (*Sphk1/2*^*ΔLEC*^) pups and analyzed their mesenteric LVs. *Sphk1/2*^*ΔLEC*^ pups were phenotypically similar to *S1pr1*^*iΔLEC*^ pups and had significantly fewer mesenteric LVs (Fig. 6 A). Analysis of the ears of 3-mo-old *Sphk1/2*^*ΔLEC*^ mice revealed fewer dermal LVs (Fig. 6 B). Additionally, the dermal lymphatic vascular density was increased in *Sphk1/2*^*ΔLEC*^ mice. These data confirmed our hypothesis and showed that LV development is regulated by S1P produced by LECs, which could be activating S1PR1 signaling in an autocrine or paracrine manner. LVVs and venous valves of *Sphk1/2*^*ΔLEC*^ mice were not analyzed, as these valves are exposed to S1P derived from blood endothelial cells and hematopoietic cells.

To determine if LEC-derived S1P inhibits TLO formation, we analyzed the mesenteries of 1-year-old S1PR1-GS and S1PR1-GS*; Sphk1/2*^*ΔLEC*^ mice. The lymphatic vessels of control mice were GFP^+^, demonstrating that S1PR1 signaling is active in LECs (Fig. 6 C, arrows). In contrast, GFP expression was downregulated in the lymphatic vessels of *Sphk1/2*^*ΔLEC*^ mice (Fig. 6, C and D), as previously demonstrated (Del Gaudio et al., 2024; Engelbrecht et al., 2020). Additionally, numerous GFP^+^ clusters were observed in the terminal ileum of *Sphk1/2*^*ΔLEC*^ mice (Fig. 6 C, arrows). IHC for B220 and LYVE1 revealed these clusters to be TLOs (Fig. 6 E). These data indicate that cell-autonomous S1PR1 signaling in LECs inhibits TLO formation in the terminal ileum.

In summary, autocrine or paracrine S1PR1 signaling in LECs regulates LV development and inhibits TLO formation in the terminal ileum. Importantly, as TLO formation correlated with the absence of LVs or dysfunctional LVs, our finding supports the recently proposed hypothesis that LV defects could contribute to TLO formation (Czepielewski et al., 2021).

### Deletion of S1PR1 from the lymphatic vasculature does not result in epithelial dysplasia, epithelial inflammation, or microbial dysbiosis

TLOs are observed in the mesenteries of Crohn’s disease patients and in a mouse model of ileitis (Randolph et al., 2016; Czepielewski et al., 2021; Rehal and von der Weid, 2017). TLOs are thought to form due to chronic inflammation, although this possibility has not been tested (Ruddle, 2014). Whether TLOs can promote or aggravate the disease by causing epithelial inflammation and tissue damage is also not known.

**Figure 7.**
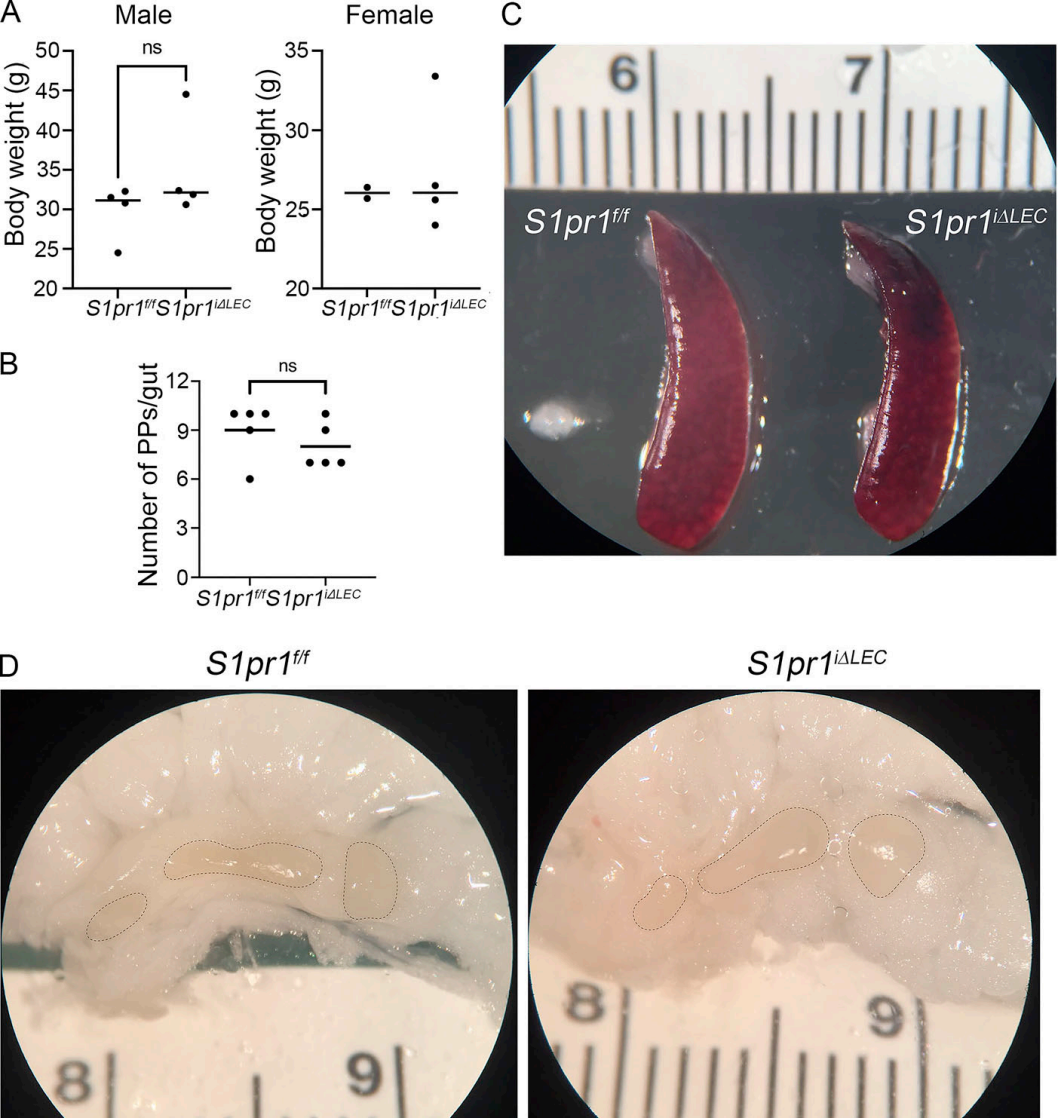
***S1pr1*** ^***iΔLEC***^ **mice did not have obvious characteristics of inflammatory disease. (A)** 10-mo-old *S1pr1*^*f/f*^ (TM@P1–7) and *S1pr1*^*iΔLEC*^ (TM@P1–7) littermates had comparable body weights irrespective of sex. **(B)** The number of Peyer’s patches in the guts (duodenum to cecum) of 10-mo-old *S1pr1*^*f/f*^ (TM@P1–7) and *S1pr1*^*iΔLEC*^ (TM@P1–7) littermates was counted and found to be comparable. **(C and D)** Spleen (C) and mesenteric LNs (D) of 10-mo-old *S1pr1*^*f/f*^ (TM@P1–7) and *S1pr1*^*iΔLEC*^ (TM@P1–7) littermates were comparable in size and shape. Statistics: (A) Each dot in the graph represents an individual animal. *n* = 4 male *S1pr1*^*f/f*^, *n* = 4 male *S1pr1*^*iΔLEC*^, *n* = 2 female *S1pr1*^*f/f*^, and *n* = 4 female *S1pr1*^*iΔLEC*^. Statistical significance was calculated for males using Mann–Whitney test; (B) *n* = 4 males and 1 female per genotype. Statistical significance was calculated using Mann–Whitney test; (C and D) images are representative of *n* = 3 animals/genotype/sex.

**Figure 8.**
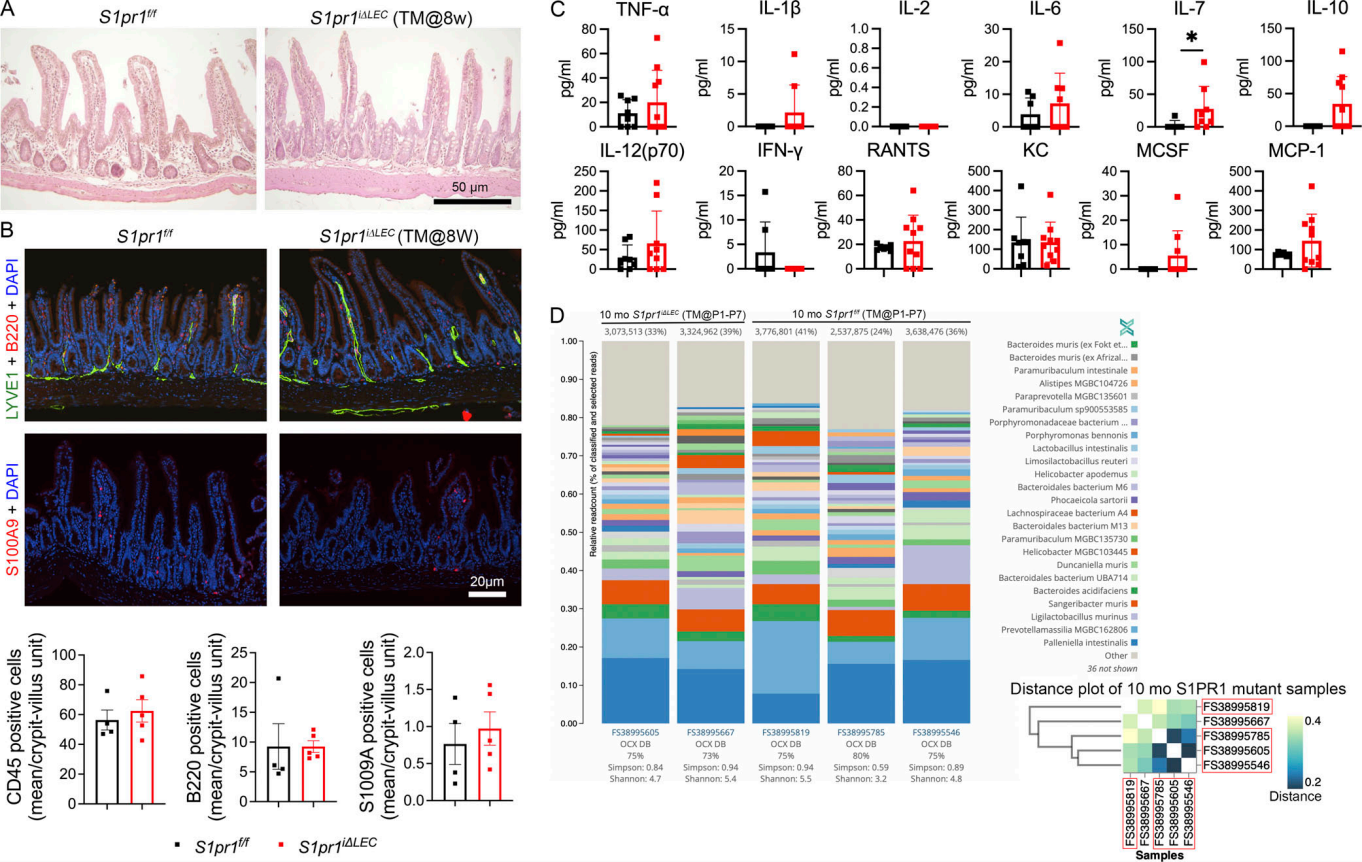
***S1pr1*** ^***iΔLEC***^ **mice do not develop epithelial dysplasia, ileal inflammation, or microbial dysbiosis. (A and B)** The ileum of *S1pr1*^*f/f*^ and *S1pr1*^*iΔLEC*^ mice (TM@8w) were sectioned and analyzed by H&E (A) or IHC for the indicated markers (B). **(A)** The intestinal epithelium of *S1pr1*^*iΔLEC*^ mice appeared to be indistinguishable from control samples with no obvious infiltration of immune cells. **(B)** The same number of CD45^+^ hematopoietic cells, B220^+^ B cells, and S100A9^+^ neutrophils were observed in the control and *S1pr1*^*iΔLEC*^ mice. **(C)** The serum from *S1pr1*^*f/f*^ and *S1pr1*^*iΔLEC*^ mice was analyzed by multiplex ELISA for inflammatory cytokines. IL-7 was modestly increased in the *S1pr1*^*iΔLEC*^ mice. No significant differences were observed between the control and *S1pr1*^*iΔLEC*^ mice for the other cytokines. **(D)** The fecal pellets of *S1pr1*^*f/f*^ and *S1pr1*^*iΔLEC*^ mice were analyzed by shotgun metagenomic analysis. The numbers on top of the graphs indicate the number of bacterial reads. The same information is provided within brackets as a percentage of total reads. The graphs indicate the relative abundance of the bacterial species. No obvious change was observed between the *S1pr1*^*f/f*^ and *S1pr1*^*iΔLEC*^ mice. The inset is a hierarchical clustering of the same samples with the co-housed mice within red boxes. Three of the four co-housed mice appear to be closer to each other irrespective of their genotype. Statistics: (A and B) *n* = 4 control and *n* = 5 *S1pr1*^*iΔLEC*^ mice (TM@8w). Two to four sections per mice were analyzed, and the number of cells per crypt-villus unit was calculated, and graphs were plotted as mean ± SEM. Statistical significance was calculated using unpaired *t* test for CD45 and S100A9 and Mann–Whitney test for B220; (C) *n* = 8 control and *n* = 10 *S1pr1*^*iΔLEC*^ mice (TM@8w). Graphs were plotted as mean ± SD. Significance was determined using unpaired *t* test with Welch’s correction for MCP-1 and RANTES and Mann–Whitney test for the other cytokines. **(D)***n* = 2 male *S1pr1*^*iΔLEC*^ (TM@P1–7) and *n* = 3 TM-treated *S1pr1*^*f/f*^ control littermates. In the distance plot, cohoused mice are within the red box. *P < 0.05.

The body weight, number of Peyer’s patches, spleen size, and mesenteric LN size of 1-year-old *S1pr1*^*iΔLEC*^ mice were indistinguishable from those of control littermates (Fig. 7). H&E staining did not reveal differences in epithelial morphology of the terminal ileum of control and *S1pr1*^*iΔLEC*^ mice (Fig. 8 A), nor did IHC reveal any obvious increase in the infiltration of S100A9^+^ neutrophils or B220^+^ B cells in the ileum of mutant mice (Fig. 8 B). Of a panel of cytokines measured in the serum of *S1pr1*^*iΔLEC*^ mice, we only observed a modest increase in IL-7 (Fig. 8 C). Although a trend to an increase was also observed in 11 other inflammatory cytokines, including TNFα and IL-1β, these changes were not significant (Fig. 8 C).

GWAS studies have implied that an abnormal immune response to commensal bacteria is the primary cause of Crohn’s disease (Belkaid and Hand, 2014; Rivas et al., 2011; Caprilli, 2008). Crohn’s disease is associated with microbial dysbiosis, in which a reduction in beneficial organisms (e.g., *Faecalibacterium prausnitzii*, of the phyla Firmicutes) and an expansion of pathological microorganisms (e.g., *Escherichia coli*, of the phyla Proteobacteria) is observed (Belkaid and Hand, 2014; Sokol et al., 2009). Microbiota dysbiosis was also observed in mouse models of ileitis (Lamas et al., 2016; Petnicki-Ocwieja et al., 2009; Schaubeck et al., 2016). We performed shotgun metagenomic analysis of fecal pellets from the terminal ileum of 12-mo-old *S1pr1*^*iΔLEC*^ mice and littermate controls. No striking differences were observed in the bacterial contents of the mutants when compared with their control littermates (Fig. 8 D). Thus, microbial dysbiosis is not observed in *S1pr1*^*iΔLEC*^ mice.

In summary, *S1pr1*^*iΔLEC*^ mice do not have the characteristics of ileitis. Thus, TLOs in the mesentery of *S1pr1*^*iΔLEC*^ mice are a sign of subclinical local inflammation that does not result in systemic inflammation, epithelial damage, or microbial dysbiosis.

### S1PR1 regulates cytoskeletal organization, OSS response, and the expression of valve-regulatory genes

OSS can enhance the expression of molecules such as the transcription factor FOXC2 and the gap junction molecule CX37, both of which are critical for lymphatic vessel maturation and LV development (Sabine et al., 2012; Geng et al., 2021; Sweet et al., 2015; Kanady et al., 2011). LECs require the ion channel PIEZO1 and the adherens junction molecule VE-cadherin for sensing OSS and activating the expression of valve-regulatory molecules (Choi et al., 2019; Yang et al., 2019). However, the mechanisms by which LECs sense and transduce OSS are not fully understood.

S1PR1 can regulate the cytoskeleton, stabilize adherens junctions, and mediate shear stress responses in blood endothelial cells (Jung et al., 2012; Lee et al., 1999). We previously showed that S1PR1 is necessary for cytoskeletal organization in primary human LECs (HLECs) (Geng et al., 2020). We also showed that S1PR1 can regulate the laminar shear stress response in HLECs (Geng et al., 2020). Hence, we hypothesized that S1PR1 signaling regulates the expression of valve-regulatory molecules in response to OSS.

**Figure 9.**
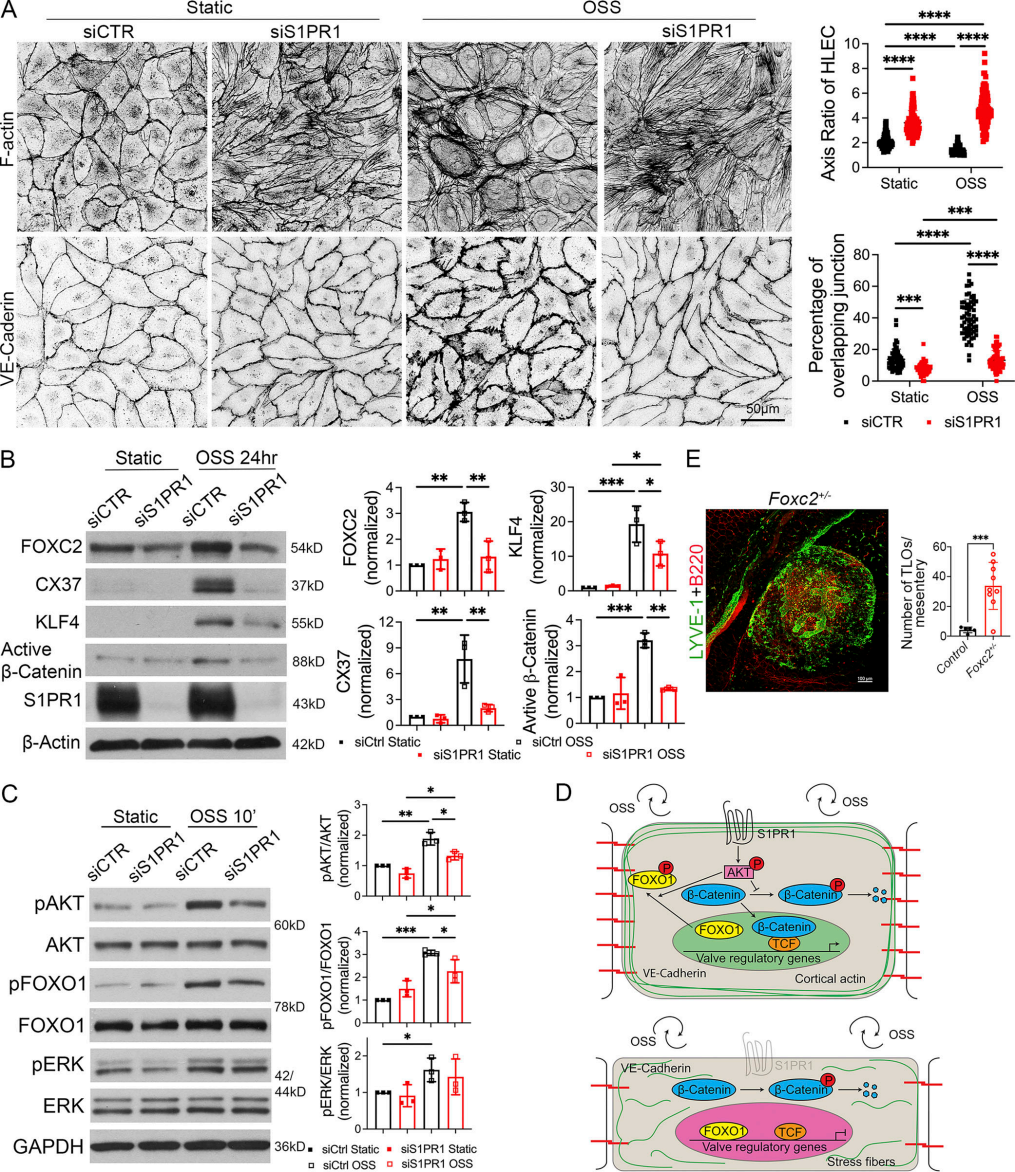
**S1PR1 regulates OSS response and the expression of valve regulatory genes in HLECs. (A)** HLECs were transfected with siControl or siS1PR1 and grown for 24 h under static conditions to knockdown S1PR1. Subsequently, cells were cultured under static or OSS for 24 h. Cells were immunostained for F-actin or VE-cadherin. F-actin was primarily located along the cell wall (cortical actin) of control cells under both static and OSS conditions. Control HLECs became more spherical, and cortical actin expression appeared to be increased by OSS. In contrast, siS1PR1-transfected HLECs appeared elongated and had increased expression of stress fibers. The percentage of VE-cadherin^+^ overlapping cell junctions was increased by OSS, and this enhancement was abolished by siS1PR1. **(B)** HLECs were cultured as described above, and western blotting was performed for the indicated proteins. OSS induced the expression of the shear stress-responsive transcription factor KLF4 and the valve-regulatory molecules active β-catenin, FOXC2, and CX37. Knockdown of S1PR1 significantly inhibited the expression of these molecules. **(C)** HLECs were transfected with siControl or siS1PR1 and grown for 48 h under static conditions to knockdown S1PR1. Subsequently, cells were cultured under static or OSS for 10 min. Cell lysates were western blotted for the indicated antibodies, and quantified. pAKT, pERK, and pFOXO1 were upregulated by OSS. siS1PR1 significantly downregulated the expression of pAKT and pFOXO1. **(D)** Schematic summary of OSS response in LECs. S1PR1 preserves VE-cadherin and cortical actin and promotes the phosphorylation of AKT. Phosphorylated AKT promotes the phosphorylation and nuclear exclusion of FOXO1 and prevents the phosphorylation and degradation of β-catenin. The later two processes are likely responsible for the expression of valve-regulatory molecules FOXC2 and CX37 (encoded by *GJA4*) and the shear-stress responsive transcription factor KLF4. In the absence of S1PR1, LECs lose VE-cadherin, gain stress fibers, become elongated, and do not upregulate valve-regulatory genes or KLF4 in response to OSS. **(E)** The mesenteric tissues from 1-year-old control and *Foxc2*^*+/−*^ mice were analyzed by IHC for the indicated markers to identify and quantify TLOs. A representative TLO from a *Foxc2*^*+/−*^ mouse is shown. A significantly higher number of TLOs were observed in the *Foxc2*^*+/−*^ mice. Statistics: (A) The axis was measured in 30 cells, and the junction was analyzed in 20 cells in a single field from each of the three experiments. Each dot represents one cell in the graphs; (B and C) the blot is representative of three independent experiments. The data from all experiments were used to prepare the graphs; (E) each dot in the graph indicates an individual mouse. *n* = 5 controls, *n* = 9 *Foxc2*^*+/−*^ mice. The graphs are shown as mean ± SD. Two-way ANOVA with Tukey’s post hoc test (A–C) and unpaired *t* test with Welch’s correction (E) were performed to determine statistical significance. *P < 0.05; **P < 0.01; ***P < 0.001; ****P < 0.0001. Source data are available for this figure: SourceData F9.

We performed IHC for the expression of actin and VE-cadherin in siControl- and siS1PR1-transfected HLECs grown under static or OSS conditions. As reported previously, control HLECs became more spherical in response to OSS and had thicker cortical actin fibers (Sabine et al., 2012; Sabine et al., 2015). Cell–cell junctions changed from linear junctions under static conditions to overlapping junctions (Fig. 9 A). In contrast, siS1PR1-transfected HLECs were elongated in shape under both static and OSS conditions (Fig. 9 A). Stress fibers that crisscrossed the cytoplasm were the predominant type of actin that was observed. Furthermore, siS1PR1-transfected HLECs maintained linear cell–cell junctions despite exposure to OSS. These results demonstrated that S1PR1 is necessary for cytoskeletal architecture and adherens junction assembly in OSS-exposed HLECs.

We transfected HLECs with control siRNA or siS1PR1 and cultured them for 24 h under static or OSS conditions. Cells were lysed, and western blotting was performed to quantify the expression of FOXC2 and CX37. OSS induced the expression of FOXC2 and CX37 in control HLECs, as reported previously (Sabine et al., 2012). Knockdown of S1PR1 significantly downregulated the expression of FOXC2 and CX37 (Fig. 9 B). We previously showed that OSS enhances Wnt/β-catenin signaling, which is necessary for valve development and FOXC2 expression (Cha et al., 2016). Accordingly, OSS enhanced the expression of active β-catenin (Fig. 9 B). In contrast, active β-catenin was not upregulated in siS1PR1-transfected HLECs (Fig. 9 B). Thus, S1PR1 regulates Wnt/β-catenin signaling and the expression of valve regulatory molecules in response to OSS.

Acute OSS promotes AKT phosphorylation in HLECs in a VE-cadherin–dependent manner (Yang et al., 2019). In turn, pAKT phosphorylates and inhibits the transcription factor FOXO1 to derepress FOXC2 (Scallan et al., 2021). We knocked down S1PR1 in HLECs and cultured them under static or OSS conditions for 10 min. Western blotting revealed that OSS enhanced the phosphorylation of AKT and ERK in control HLECs (Fig. 9 C). Knockdown of S1PR1 significantly reduced the phosphorylation of AKT and FOXO1 in response to OSS (Fig. 9 C).

In summary, S1PR1 regulates cytoskeletal and adherens junction organization in HLECs (Fig. 9 D). S1PR1 also promotes the phosphorylation of AKT in response to OSS. The OSS-S1PR1-pAKT pathway results in the expression of valve regulatory molecules. Phosphorylated AKT phosphorylates FOXO1 and triggers its nuclear exclusion. Phosphorylated AKT is also likely responsible for the stabilization of transcriptionally active β-catenin through the inhibition of GSK3. KLF4 might be regulated by Wnt/β-catenin signaling, as previously reported in blood endothelial cells (Cowan et al., 2010).

### Foxc2-heterozygous mice develop TLOs in the mesentery

As reported above, S1PR1 regulates the expression of several genes that are necessary for LV development, among which FOXC2 is a central player. LVs do not develop in mice lacking FOXC2 (Petrova et al., 2004). Heterozygous loss-of-function mutations in *FOXC2* are associated with lymphedema-distichiasis syndrome (LDS) (Brice et al., 2002; Bell et al., 2001). LDS patients have incompetent venous valves (Mellor et al., 2007). Whether their LVs are also defective is not known due to the difficulties associated with imaging their activity. *Foxc2*^*+/−*^ mice, used as a model of LDS, possess ∼50% fewer mesenteric LVs (Scallan et al., 2021). Slight leakage was observed in the remaining valves (Scallan et al., 2021). Approximately 50% of LDS patients develop “multiple small nodules” in the mesentery (Brice et al., 2002). Whether these nodules are TLOs is not known, although an excessive number of LNs were reported to develop throughout the body of *Foxc2*^*+/−*^ mice (Kriederman et al., 2003). Hence, we analyzed the mesenteries of *Foxc2*^*+/−*^ mice and determined that they indeed have TLOs in the terminal ileum (Fig. 9 E). These data indicate that FOXC2 is a physiologically relevant target of S1PR1 during both LV development and TLO formation and suggest that LV defects could lead to TLO formation.

## Discussion

**Figure 10.**
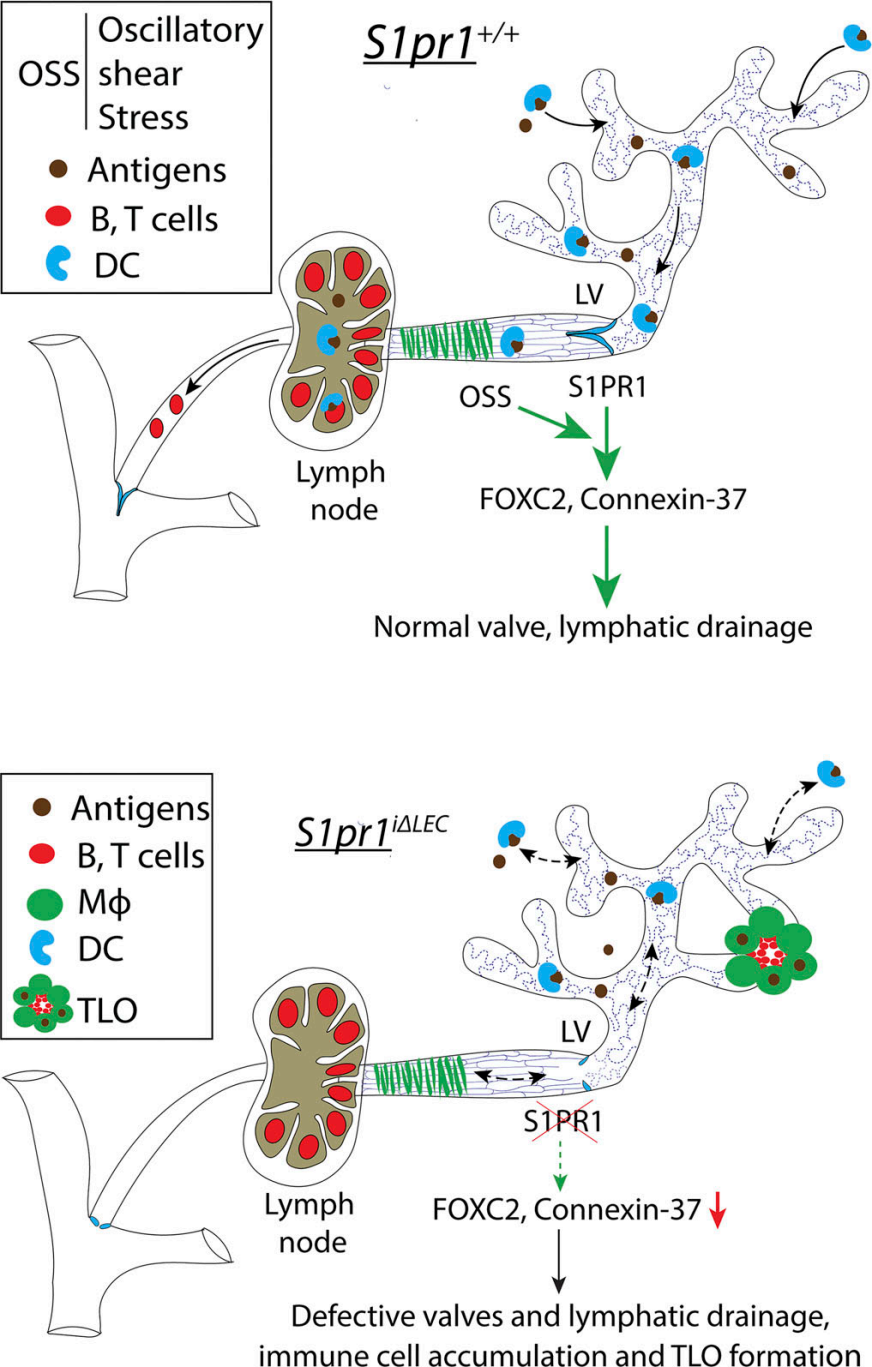
**Working model.** LVs are necessary for proper immune cell drainage to the LNs. S1PR1 regulates LV development by enhancing the expression of FOXC2 and CX37 in response to OSS. The loss of S1PR1 results in reduced lymph flow and TLO formation.

We report several new discoveries in this work: (1) S1PR1 signaling is a novel regulator of LV development; (2) differences exist between mesenteric LVs along the proximal to distal axis; (3) TLOs can form in the absence of severe inflammation or tissue damage; and (4) LV defects can result in TLO formation. A schematic working model based on our findings is presented in Fig. 10.

### S1PR1 and LV development

OSS induces the expression of valve-regulatory molecules, such as FOXC2 and CX37 in vitro (Sabine et al., 2012). OSS is sensed and transduced by the mechanosensory ion channel PIEZO1 (Choi et al., 2019). The endothelial adherens junction molecule VE-cadherin is necessary for activating Wnt/β-catenin signaling and AKT phosphorylation in response to OSS (Yang et al., 2019). In turn, β-catenin forms a complex with PROX1 to promote FOXC2 expression, and pAKT promotes FOXC2 expression by inhibiting FOXO1 (Scallan et al., 2021; Cha et al., 2018; Niimi et al., 2021). In this work, we have identified S1PR1 as a molecule that is necessary for the phosphorylation of AKT and FOXO1 in response to OSS and for the expression of the valve-regulatory molecules active β-catenin, FOXC2, and CX37 (Fig. 9 D). S1PR1 likely regulates the OSS response through the cytoskeletal architecture and adherens junction complex.

We previously showed that S1PR1 is not necessary for laminar shear stress-mediated phosphorylation of AKT (Geng et al., 2020). Our observations suggest that S1PR1 regulates LSS and OSS through distinct mechanisms. We speculate that these differences could be due to specific interaction of S1PR1 signaling with other signaling pathways (VEGFR2, VEGFR3, other receptor tyrosine kinases, integrins, or ion channels) under LSS or OSS conditions. Our in vivo work also suggests that S1P is necessary for LV development and that it activates S1PR1 signaling in an autocrine or paracrine manner. Additional studies are necessary to delineate these relationships.

### Heterogeneity of mesenteric LVs

Previous publications have only evaluated LV formation and function in the duodenum or jejunum (Castorena-Gonzalez et al., 2020; Lapinski et al., 2017; Sabine et al., 2015; Sabine et al., 2018; Scallan et al., 2021). We have identified some intriguing peculiarities in the LVs that drain the ileum when compared with those that are in the proximal sections of the GI tract. Neonatal deletion of S1PR1 results in a reduced number of mesenteric valves, especially in the ileal mesentery. Although LVs were reduced in number also in the proximal GI sections of the mutant mice, the remaining LVs did not degenerate in adulthood. However, LVs in the terminal ileum of mutant mice are functionally defective. This defect occurs even if *S1pr1* is deleted from adult mice. We currently do not know the reason for these regional differences in LV development and function. We speculate (below) that the microbial antigens and cytokines that are generated in response to these antigens could compromise LV function in the terminal ileum. S1PR1 signaling appears to be necessary to minimize the impact of these damaging factors.

### Mechanisms of TLO formation

TLOs were not observed in the skin or proximal GI tract of *S1pr1*^*iΔLEC*^ mice. The ileocecal junction of the GI tract, where the TLOs were predominantly observed, has the highest density of microbiota in the foregut (Ivanov et al., 2009). Relevant to our work, dendritic cells engulf infiltrating bacteria, migrate from the gut to the mesenteric LNs and Peyer’s patches via lymphatic vessels, and activate T cells and IgA-secreting B cells (Belkaid and Hand, 2014; Hooper et al., 2012). These activated lymphocytes exit the LNs through lymphatic vessels, enter the bloodstream, and return to the gut to restrict pathological organisms and maintain microbial homeostasis. We speculate that due to LV defects *S1pr1*^*iΔLEC*^, *Sphk1/2*^*ΔLEC*^, and *Foxc2*^*+/−*^ mice are unable to build a systemic immune response against microbiota in the mesenteric LNs (Fig. 10). Consequently, TLOs develop in the terminal ileum to respond against microbial antigens locally and prevent microbial dysbiosis.

### Limitations of the study

Several molecules, including S1PR1, that are necessary for valve development in vivo are also important for OSS response in vitro. Nevertheless, whether lymphatic vessels experience OSS during LV development and whether OSS is essential for LV development in vivo remain unknown. Therefore, we cannot exclude additional mechanisms, for example, response to ECM stiffness, through which S1PR1 might regulate LV development.

We speculate that S1PR1 inhibits TLO formation by regulating LV development and function and thus promoting efficient lymphatic drainage. In support of this possibility, TLOs were found in *Foxc2*^*+/−*^ mice that have LV defects. Additionally, TNFα can inhibit the expression of valve-regulatory molecules, and TLOs frequently form near the LVs of *Tnf*^*+/ΔARE*^ mice (Czepielewski et al., 2021). We predict that other mouse models and humans with compromised lymphatic drainage in the ileum due to LV or lymphatic vessel defects will also have TLOs in this location. However, we cannot rule out alternative reasons for TLO formation. For example, S1PR1 signaling can antagonize TNFα-induced inflammation in blood endothelial cells (Galvani et al., 2015). S1PR1 can also inhibit the expression of pro-inflammatory molecules such as *Irf8*, *Lbp*, *Il7*, *Il33*, *Ccl21*, and *Tnfaip8l1* in LECs of mice (Engelbrecht et al., 2020). S1PR1 inhibits the expression of P-selectin in HLECs to prevent CD4^+^ T cell differentiation (Kim et al., 2023). Further experiments are necessary to determine if the S1PR1/FOXC2 axis inhibits TLO formation by antagonizing LEC inflammation.

### Clinical relevance

IBD is a chronic inflammatory condition that affects the gastrointestinal tract and has substantial effects on all aspects of life (Burisch et al., 2013). IBDs affect 1.4 million individuals in North America and 2.2 million individuals in Europe (Loftus, 2004). The two main forms of IBD are ulcerative colitis and Crohn’s disease, both of which have an incidence of 3–20 per 100,000 in the United States (Saleh and Elson, 2011). Crohn’s disease is treated with anti-inflammatory and immunosuppressive drugs, such as anti-TNF therapies, antibiotics, and surgery (Van Assche et al., 2009). These treatments manage symptoms but never cure the disease, which causes episodic, lifelong illness. Thus, a better understanding of the etiopathology and mechanisms of Crohn’s disease is needed for the development of novel therapeutics (Saleh and Elson, 2011). The presence of TLOs in the terminal ileum is one of the defining characteristics of Crohn’s disease (Randolph et al., 2016; McNamee et al., 2013; Fujimura et al., 1996; Duijvestijn et al., 1988; Kaiserling, 2001). Importantly, TLOs are thought to perpetuate inflammation by increasing immune cell retention and exacerbate tissue damage (McNamee et al., 2013). Our discovery that *S1pr1*^*iΔLEC*^ mice spontaneously develop TLOs in the terminal ileum without obvious tissue damage and inflammation appears to challenge this paradigm.

*Tnf* ^*+/ΔARE*^ mice weigh significantly less than their control littermates, have dysplastic intestinal epithelium, and have reduced survival (Rehal and von der Weid, 2017; Czepielewski et al., 2021). In contrast, despite the presence of TLOs, *S1pr1*^*iΔLEC*^ mice do not display changes in body weight, epithelial morphology, or overall survival. It is possible that the TLOs in *Tnf*^*+/ΔARE*^ mice are phenotypically distinct and more inflammatory when compared with those that are observed in *S1pr1*^*iΔLEC*^ mice. The systemic effects of TNFα overexpression in *Tnf*^*+/ΔARE*^ mice, such as poorly developed adipose tissue, cachexia, and their potential effects on the intestinal epithelium, may also contribute to the phenotype (Beutler and Cerami, 1986). Thus, the *S1pr1*^*iΔLEC*^ mice could provide a complementary model to understand the significance of TLOs in Crohn’s disease. We speculate that the TLOs in *S1pr1*^*iΔLEC*^ mice are a sign of subclinical inflammation that does not cause tissue damage. Inflammatory triggers such as bacterial infections, food allergens, or risk alleles might trigger an aggravated response by the immune cells in the TLOs, resulting in an IBD-like phenotype.

Finally, TLOs are important in the pathophysiology of several autoimmune diseases and cancer. Hence, it will be important to determine if *S1pr1*^*iΔLEC*^ mice can develop TLOs in other organs, such as the lungs, if appropriate antigens are present, such as during influenza or COVID-19 infection or chronic smoking. Whether tumor antigens can trigger TLO formation in *S1pr1*^*iΔLEC*^ mice should also be tested. The effect of mesenteric tissue TLOs on metabolic disorders such as obesity should also be investigated. Nearly a dozen FDA-approved drugs are used for inhibiting S1PR1 and treating a variety of autoimmune diseases, including multiple sclerosis and IBD (Cartier and Hla, 2019). Our finding that the deletion of S1PR1 in LECs can result in the formation of TLOs raises both hope and concern regarding these drugs. On the one hand, these drugs could be repurposed to trigger the formation of TLOs and potentiate immune checkpoint therapies for cancer. On the other hand, the formation of TLOs might compromise and even reverse the effectiveness of these drugs in treating autoimmune diseases. These possibilities must be investigated carefully.

## Materials and methods

### Mice

*Lyve1-Cre* (Pham et al., 2010), *S1pr1*^*flox*^ (Allende et al., 2003), and S1PR1-GS (Kono et al., 2014) mice were described previously and were purchased from Jackson Laboratory (catalog numbers 012601, 019141, and 026275, respectively). *Sphk1*^*flox*^ and *Sphk2*^*+/−*^ mice were reported previously (Pham et al., 2010). Tg(Prox1-CreERT2) was a gift from Dr. Taija Makinen (Uppsala University) (Bazigou et al., 2011). *Vegfr3*^*+/EGFP*^ mice were a gift from Dr. Hirotake Ichise (University of Ryukyus) (Ichise et al., 2010). Mice at Oklahoma Medical Research Foundation (OMRF) and Institut National de la Santé et de la Recherche Médicale were maintained in C57BL6:NMRI and C57BL6: 129/SvJ mixed backgrounds, respectively. All the mice were fed standard chow diet. TM was used for deleting S1PR1 in a time-specific manner. For early postnatal deletion, P1 S1pr1^iΔLEC^ and control littermate pups were fed 1 μl of 20 mg/ml TM, P3 pups 3 μl, and so on until P7. For adult deletion, 8-wk-old mice were treated with TM by oral gavage (100 μg per gram of body weight) for three consecutive days. TM stock was prepared by dissolving 200 mg of TM (T5648; Sigma-Aldrich Marketing, Inc.) in 10 ml of peanut oil and filter sterilizing.

Study Approval: All mice were housed and handled according to the institutional IACUC protocols: OMRF protocols 22–51 and 24–30 and University of Missouri protocol 9797.

### Antibodies

The details about the antibodies that were used for IHC, western blotting, and flow cytometry are provided as tables in the supplementary materials file.

### Cells

HLECs were a gift from Dr. Donwong Choi and Young-Kwon Hong (University of Southern California) (Choi et al., 2019; Choi et al., 2017a; Choi et al., 2017b). HLECs were grown on culture dishes or glass slides coated with 0.2% gelatin and were maintained in EGM-2 EC Growth Medium-2 Bullet Kit (Lonza). All experiments were conducted using cells until passage (P) 8. HLECs were treated as potential biohazards and were handled according to institutional biosafety regulations.

### Cell treatments and analysis

#### siRNA transfection

HLECs were seeded onto modified culture dishes as described previously (dela Paz et al., 2012; Choi et al., 2019). Briefly, a 6-cm cell culture dish was adhered in the center of a 10-cm dish using nontoxic medical-grade silicone glue (A-100, medical silicone adhesive Factor II, Inc.), UV irradiated for 1 h, and dried overnight. HLECs were seeded in the donut-shaped track. After 24 h of culture (around 30% confluency), cells were transfected with siCTR (Cat# 51-01-14-03; Integrated DNA Technologies) or siS1PR1 (Cat# SI00376201; Qiagen) using Lipofectamine RNAiMax Transfection Reagent (Cat# 13778150; Invitrogen) according to the manufacturer’s instructions.

#### OSS

HLECs were seeded in culture dishes, transfected with siRNA as described above, and grown to 80–100% confluency before exposing them to OSS for either 10 min or 24 h. OSS was applied to the cells at ∼6 dyn/cm^2^ using the approach described previously (Choi et al., 2019). Briefly, the culture dishes were placed on top of bidirectional shaker (MS-NOR-30; Major Science) and horizontally rotated clockwise for ∼1 s, followed by anticlockwise rotation for ∼1 s at 100 rpm for 24 h.

#### Cell shape and cell junction analysis

The ratio of cell length to width was calculated using NIS-Elements software (Nikon). For junction analysis, a thick junction with two visible borders was identified as the overlapping junctions (Sabine et al., 2015). The ratio of the length of overlapping junction to that of the total junction was quantified using NIS-Elements software (Nikon).

### Live vessel imaging and whole-mount immunofluorescence of isolated mesenteric lymphatics

Immediately after undergoing valve functional tests, Prox1-tdTomato^+^ vessels were utilized for live confocal imaging. Mice lacking Prox1-tdTomato were stained with 25 µM Cell Tracker Green (Invitrogen) in Krebs for 45 min to 1 h while cannulated at 37°C. The vessel lumen and bath were then flushed with Krebs to remove any residual dye. The vessel was then used for live imaging. Cell Tracker Green (CM7025) was made fresh daily in DMSO at 10 mM.

The isolated and cleaned mesenteric lymphatic vessels were cannulated in “Calcium-Free” Krebs buffer to prevent movement artifacts during imaging. The vessels were imaged with an Andor Dragonfly 200 spinning disc confocal microscope on a Leica DMi8 inverted microscope equipped with a Zyla 4.2 Megapixel sCMOS camera and controlled by the Andor Fusion software. Image stacks of LVs, valve sites, or single valve leaflets were collected at 40× magnification (HCX PL APO 40×/1.10 W CORR) at 0.24-μm z-plane intervals throughout the entire vessel. 3D reconstructions were made using IMARIS software.

Following the valve functional test and live confocal imaging, vessels were used for immunostaining. While the vessels were still cannulated and pressurized, they were fixed using 1% chilled paraformaldehyde (PFA) for 20 min to maintain the vessel’s natural open state. Vessels were then fixed overnight in a 24-well dish on a rocker at 4C in 1% PFA and washed with PBS for 2–4 h the following morning. The fixed mesenteric lymphatic vessels were permeabilized with PBS supplemented with 0.1% TritonX100 for 30 min and then blocked in Blockaid buffer for 3 h. The vessels were stained with primary and secondary antibodies overnight. Vessels were washed a final time in PBS and incubated with NucBlue (R37605 Hoechst 33342; Thermo Fisher Scientific) in PBS for 5 min to stain the nuclei. Vessels were then cannulated and imaged on the Andor Dragonfly using 40× magnification, 0.24-μm step size, and a Zyla 4.2 Megapixel sCMOS camera. Image stacks were then opened in IMARIS to create a 3D reconstruction for valve visualization. The “Crop 3D” tool was used to further restrict viewing to a single LV leaflet. Display min/max values and gamma values were optimized to assist in visualizing the fluorescent signal throughout the full image stack of the vessel.

### Metagenomics analysis

Metagenomics analysis was performed on a fee-for-service basis through Transnetyx. Briefly, two fresh fecal pellets from each mouse were collected, transferred into a tube with proprietary buffer, and shipped. DNA was extracted and sequenced by Transnetyx, which was subsequently analyzed with One Codex cloud-based software.

### Multiplex enzyme-linked immunosorbent assay for cytokine

Serum cytokines were quantified using MILLIPLEX Mouse Cytokine/Chemokine Magnetic Bead Panel (EMD Millipore), and sample acquisition was performed on Bio-Plex 200 (BioRad) as per the manufacturer’s instructions (Chaaban et al., 2015).

### IHC

IHC on cryosections, vibratome sections, mesentery, and skin were performed according to our previously published protocols (Cha et al., 2016; Cha et al., 2018; Geng et al., 2016). Immunocytochemistry was performed as we described previously (Cha et al., 2016; Mahamud et al., 2019; Geng et al., 2020; Geng and Srinivasan, 2021).

For harvesting the adult mesentery, mice were euthanized by asphyxiation followed by perfusion with PBS and 4% PFA. Subsequently, the entire gut (stomach to rectum) was dissected and further fixed overnight in 2% PFA in the cold room. After washing profusely with PBS on ice, the mesenteries were dissected out from the gut and used for whole-mount IHC using the iDISCO protocol that we described previously (Geng and Srinivasan, 2021) with minor modifications. Specifically, the tissues were postfixed in 4% PFA overnight in the cold room, washed with PBS, and cleared for at least 3 days in the cold room with 1.62 M Histodenz medium and 0.1% Tween 20 (both from Sigma-Aldrich) (González-Loyola et al., 2021). Cleared tissues were mounted on slides with 1.62 M Histodenz medium, and images were taken using LSM 710 laser-scanning microscopes (Zeiss) or C2^+^ confocal (Nikon) microscopes.

### Lymphangiography

Mice were anesthetized with ketamine/xylazine (25/2.5 mg/kg, i.p.) and placed face up on a heated tissue dissection/isolation pad. The abdomen was opened, and a 2–3″ section containing the cecum and terminal ileum was pulled out and pinned, with blood supply intact, onto a semicircular base of Sylgard on the top of a transparent, water-jacketed base. The base was heated to 37°C. The preparation was continuously superfused with Krebs solution preheated to 37°C through a heating coil. The intestinal preparation was transferred to the stage of a Zeiss Axio-zoom V16 microscope for fluorescence imaging using Zeiss Zen software. The lymphatic networks in various regions of the terminal ileum were perfused with Krebs containing 1–2% FITC-dextran (Sigma-Aldrich) using sharpened micropipettes (∼40-µm tip diameter). The micropipette was positioned using a Narishige micromanipulator such that it just penetrated the intestinal wall, usually adjacent to a pin in a Peyer’s patch. The pipette was pressurized from a 10-ml glass syringe as needed to perfuse the intestinal wall lymphatic capillary network. Once that network was perfused (usually for a length of intestinal wall of 1–2 mm), pressure was maintained for 1–30 min as the dye drained into the lymphatic collectors of the mesentery. Dye perfusion was monitored by continuously recording the FITC/GFP channel of the microscope at 5 fps. Once the main collector in that region was fully perfused, or after 20–30 min had elapsed without good collector perfusion (in the case of regions with TLOs), the pipette was withdrawn, and the adjacent segment (1–2 mm away) was studied after a new micropuncture site was located. The typical procedure was to start imaging/perfusion at the last vascular arcade in the terminal ileum and then move proximally to perfuse the second and third arcades from the cecum.

Krebs solution contained: 146.9 mM NaCl, 4.7 mM KCl, 2 mM CaCl_2_·2H_2_O, 1.2 mM MgSO_4_, 1.2 mM NaH_2_PO_4_·H_2_O, 3 mM NaHCO_3_, 1.5 mM Na-HEPES, and 5 mM D-glucose (pH = 7.4), supplemented with 0.5% bovine serum albumin for isolated vessel studies.

### Protein isolation and analysis

Protein was extracted from cells by using radioimmunoprecipitation assay lysis buffer. Western blots were performed according to standard protocols. The intensities of bands were measured using ImageJ software.

### SEM

SEM was performed according to our previous protocol (Geng et al., 2016; Geng and Srinivasan, 2018). Briefly, vibratome sections were used for IHC and confocal microscopy analyses. Subsequently, the tissues were fixed in 2% glutaraldehyde in 0.1 M cacodylate buffer for 2 h. After washing profusely in PBS, the sections were postfixed in 1% osmium tetroxide in 0.1 M cacodylate buffer for 2 h and subsequently dehydrated sequentially with increasing concentrations of ethanol. The sections were further dehydrated in hexamethldisilazane and allowed to air-dry overnight. Dry sections were sputter-coated with Au/Pd particles (Med-010 Sputter Coater by Balzers-Union) and observed under Quanta SEM (FEI) at an accelerating voltage of 20 KV.

### Valve function tests

The methods for assessing back leak in isolated lymphatic collectors containing a single valve have been described in detail previously (Sabine et al., 2018; Davis et al., 2011) and documented in several recent studies of transgenic mice (Chen et al., 2020; Lapinski et al., 2017; Munger et al., 2017; Sabine et al., 2015). Mice were anesthetized with ketamine/xylazine (25/2.5 mg/kg, i.p.) and placed in the prone position on a heated tissue dissection/isolation pad. Mesenteric collectors were isolated by opening the abdomen, removing the entire small intestine, and pinning it in a Sylgard-coated dish. Individual collectors were identified, excised, and pinned to the chamber using 40-μm wire. After removing the majority of the associated fat and connective tissue, vessels containing a single valve were then transferred to a 3-ml myograph chamber containing Krebs-albumin solution and cannulated at each end with a glass micropipette (40–50 μm OD tip), pressurized slightly, and further cleaned. The chamber with attached micropipettes, pipette holders, and micromanipulators was transferred to the stage of an inverted microscope. Polyethylene tubing connected the back of each micropipette to low-pressure transducers and a computerized pressure controller, allowing independent control of Pin and Pout.

Valve function tests measured the pressure back leak through a closed valve. Starting with Pin and Pout = 0.5 cm H_2_O, and with the valve open, Pout was raised to 10 cm H_2_O, ramp-wise, over a 30-s period while Pin was held at 0.5 cm H_2_O. Normal valves closed as Pout exceeded ∼1 cm H_2_O and remained closed for the duration of the Pout ramp. In some cases, when valves appeared stiffer than normal, gentle tapping of the Pout line was used to encourage closure. Pressure back leak through the closed valve was measured with a servo-null micropipette inserted through the vessel wall on the inflow side of the vessel, which could resolve changes as small as ∼0.05 cm H_2_O. The value of Psn at the end of the ramp (Pout = 10 cm H_2_O) was used as the standard index of back leak.

### Statistical analyses

For biochemical studies, the number n refers to the number of times the experiment was performed. For histochemical analysis, *n* refers to the total number of animals included per group. Statistically significant differences were determined using unpaired *t* tests, Mann–Whitney tests, or two-way ANOVA with Tukey’s post hoc tests. Prism software was used for statistical analyses. Data are reported as mean ± SD or mean ± SEM with significance set at P < 0.05. *n* and P values for each experiment are provided in the figure legends. Western blots are performed at least three independent times. The most representative western blots are presented.

### Online supplemental material

Fig. S1 shows that the dermal LV defects in *Lyve1-Cre;S1pr1*^*−/f*^ embryos are not rescued by Vegfr3 heterozygosity. Fig. S2 demonstrates the efficiency of Tg(Prox1-CreERT2) in deleting *S1pr1* from both the proximal and distal mesenteric lymphatic vessels. Fig. S3 shows the normal expression of valve markers in the LVs that remained in *S1pr1*^*iΔLEC*^ mice. Fig. S4 shows that the LVs in the ileum, but not duodenum or jejunum, of *S1pr1*^*iΔLEC*^ mice are defective. Fig. S5 shows some of the structural defects in the LVs of *S1pr1*^*iΔLEC*^ mice. Video 1 is a live image of a fluorescent dye draining from the gut toward the mesenteric LN in a control mouse. Videos 2 and 3 show defective lymphatic drainage and the blockage of dye in nodules of *S1pr1*^*iΔLEC*^ mice. Table S1 lists all the antibodies that were used in the experiments.

## Supplementary Material

Table S1 lists all the antibodies that were used in the experiments.

SourceData F9 is the source file for Fig. 9.

## Data Availability

The metagenomics sequencing data generated for this study are available from Datadryad.org under the DOI https://doi.org/10.5061/dryad.qbzkh18vj.
