# Supplementary figures and images for: S1PR1 regulates lymphatic valve development and tertiary lymphoid organ formation in the ileum
Source: J Exp Med. 2025 Jun 24;222(9):e20241799. doi: 10.1084/jem.20241799 (PMC12187108; doi:10.1084/jem.20241799)

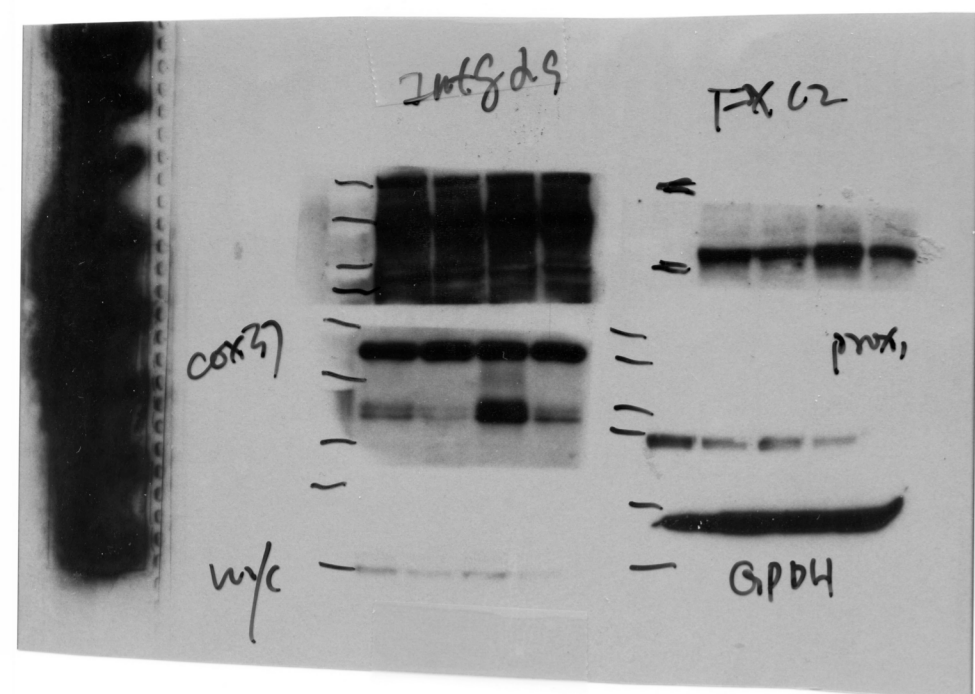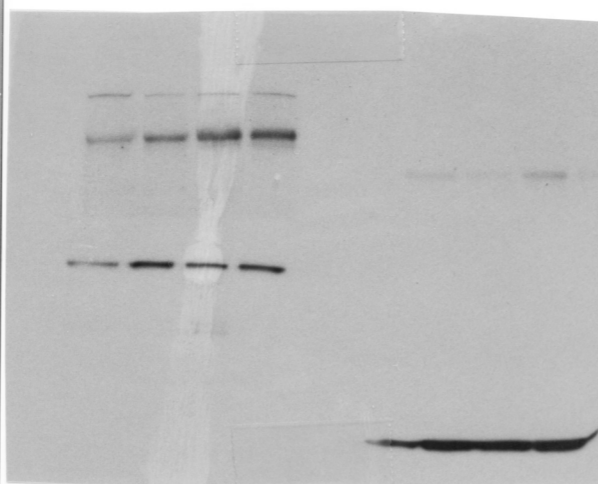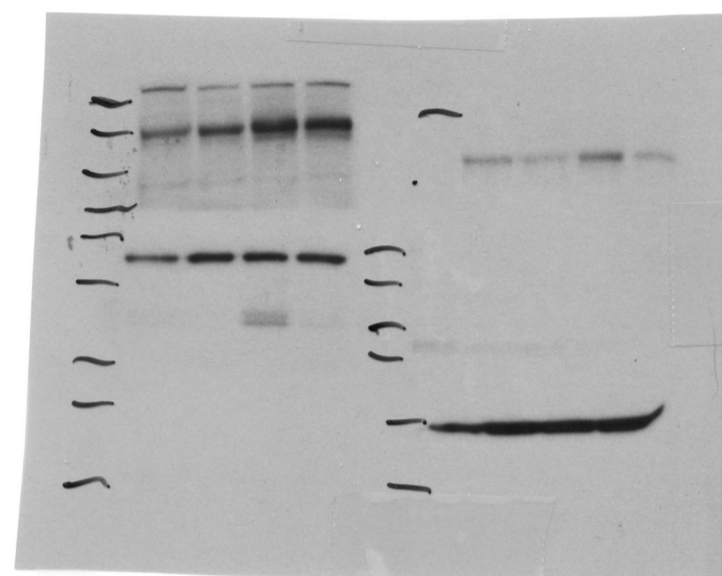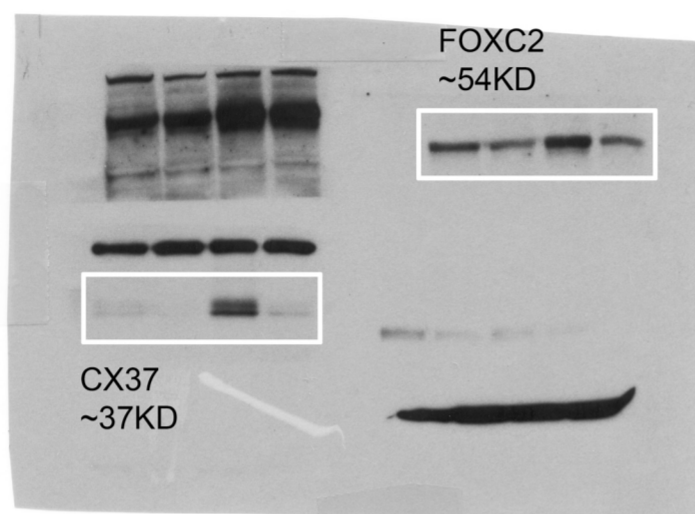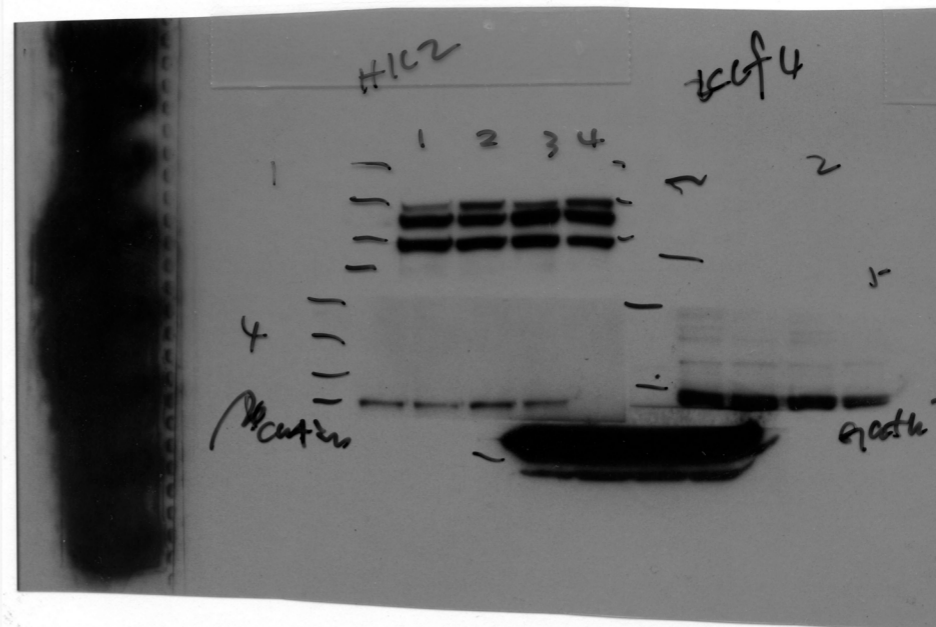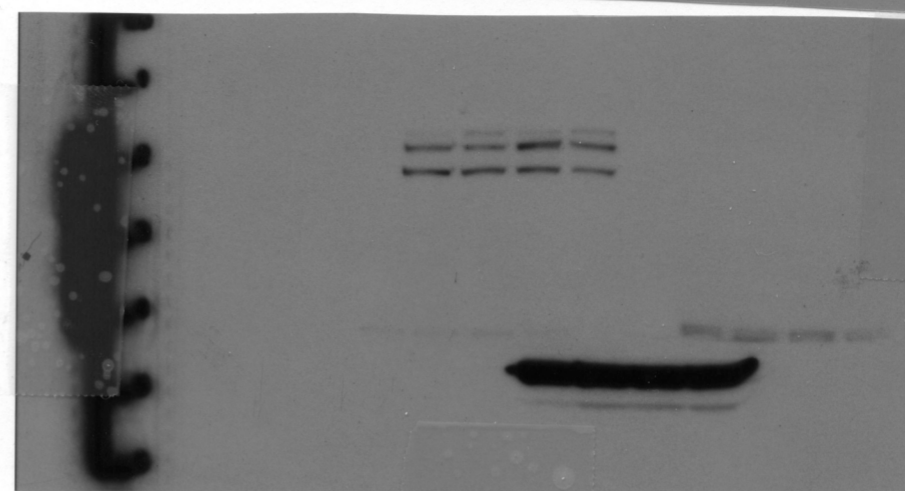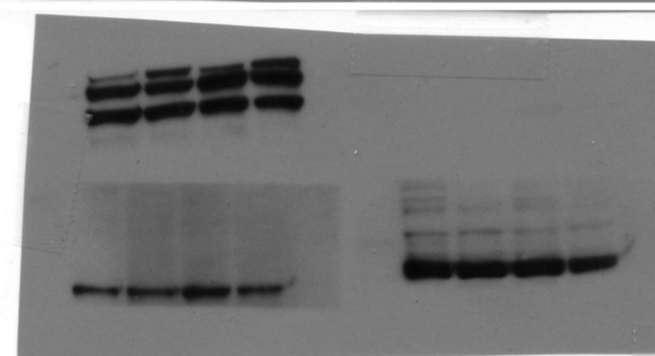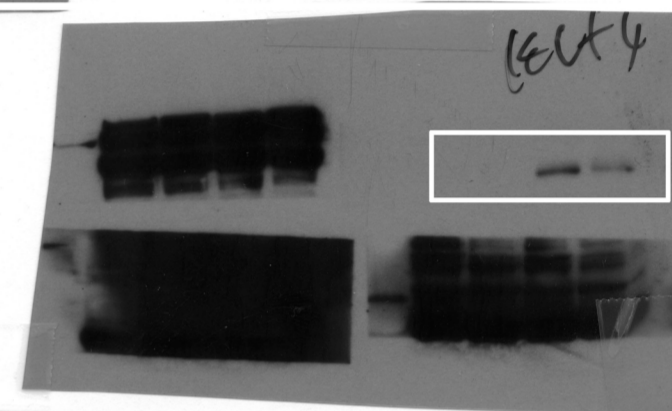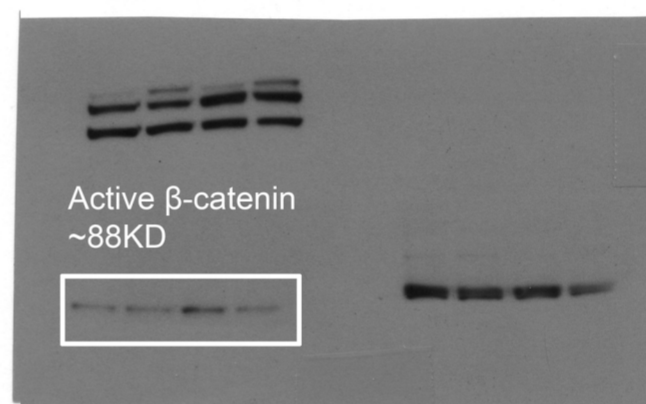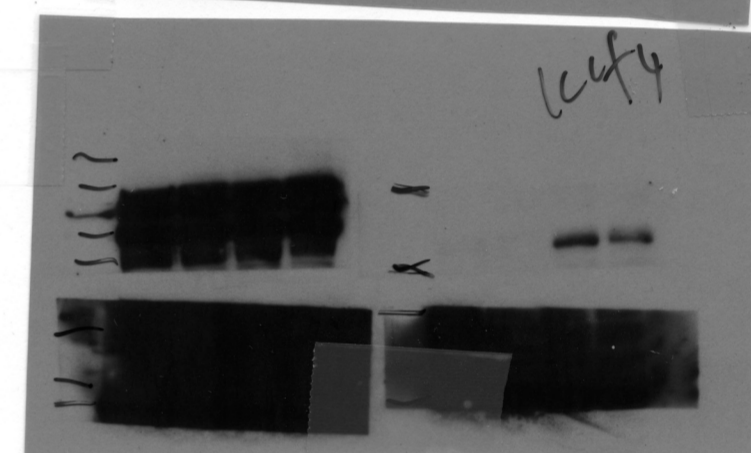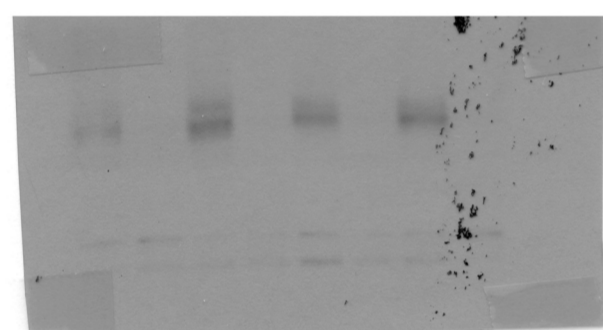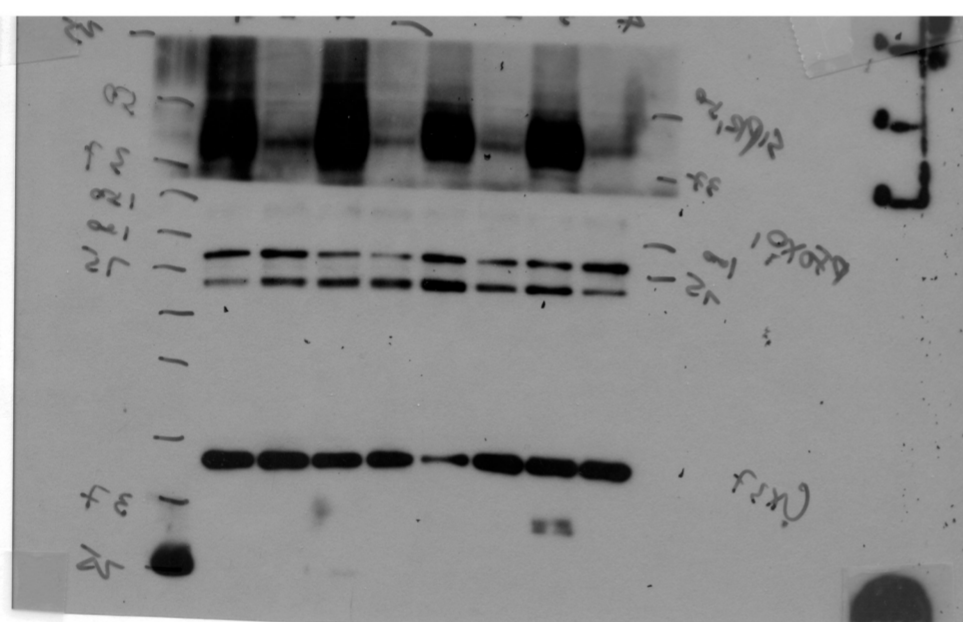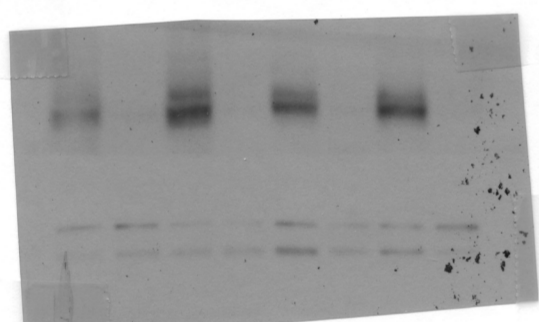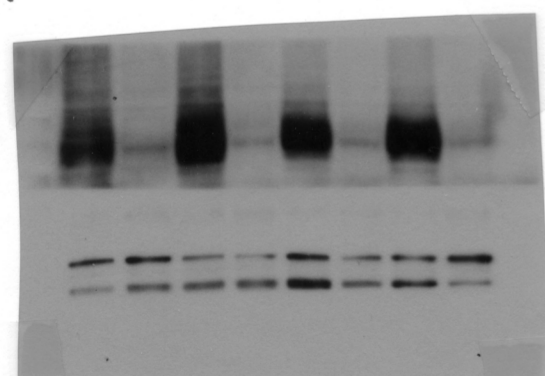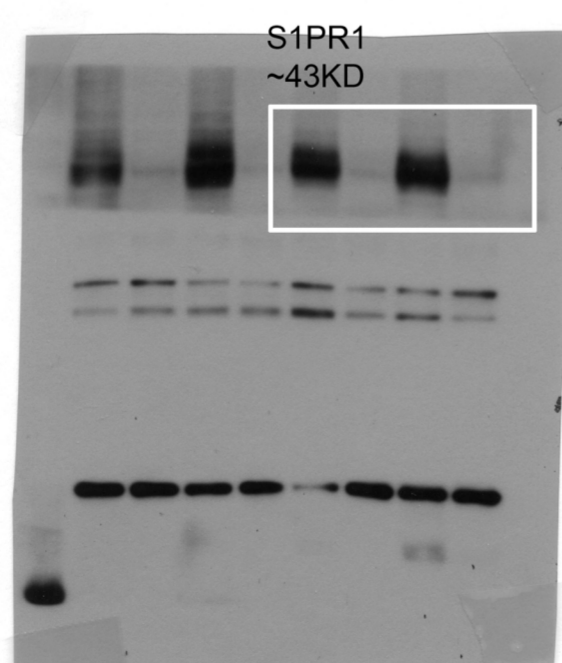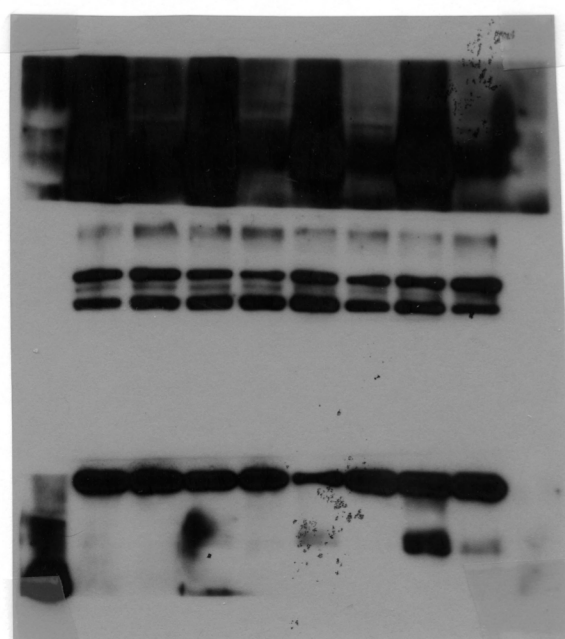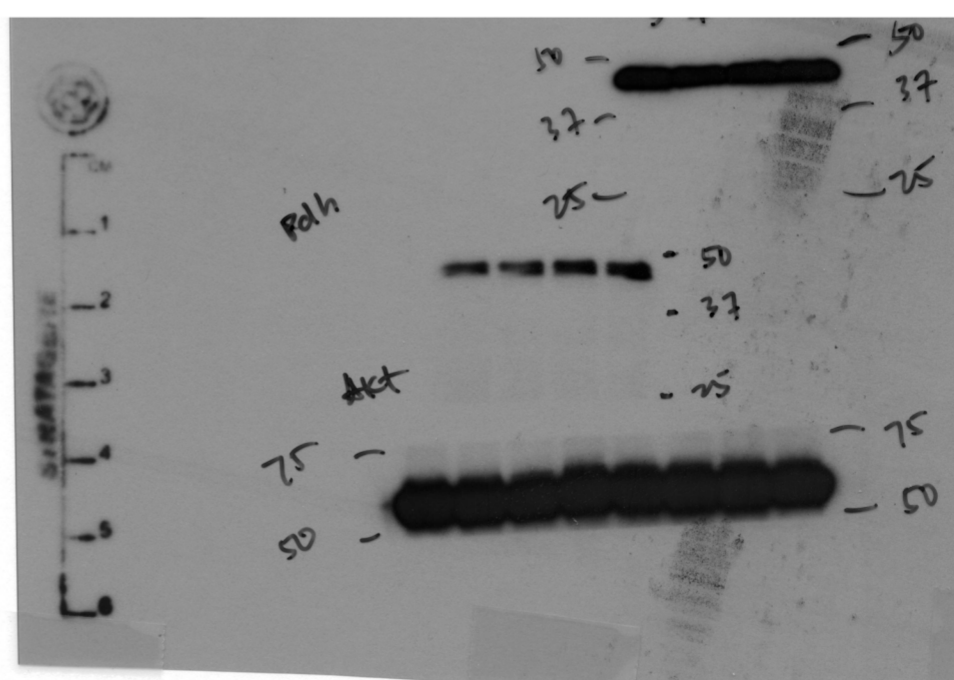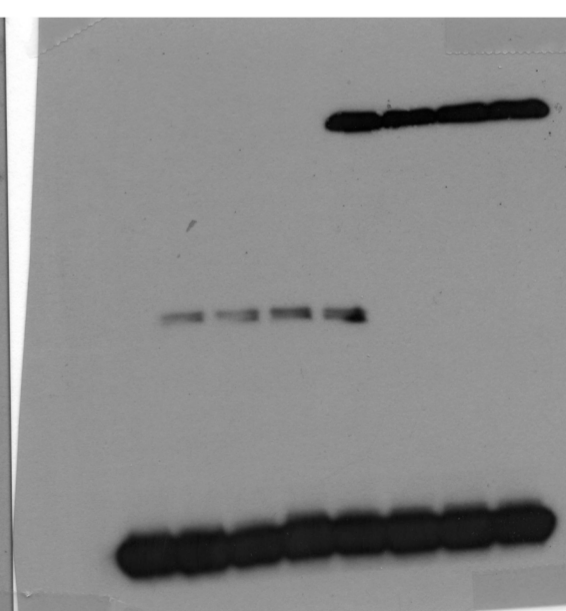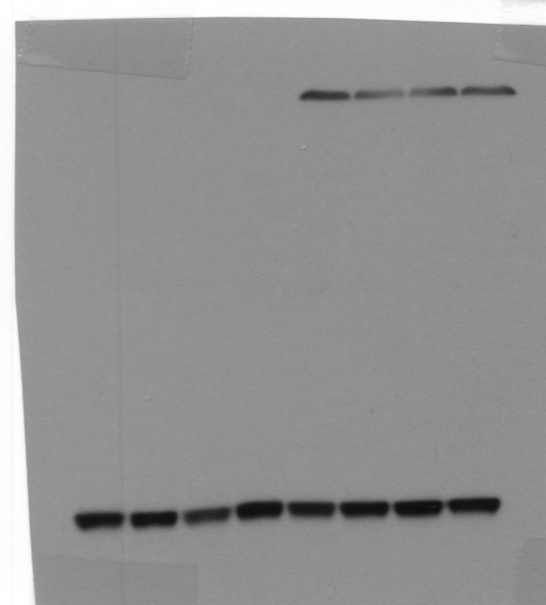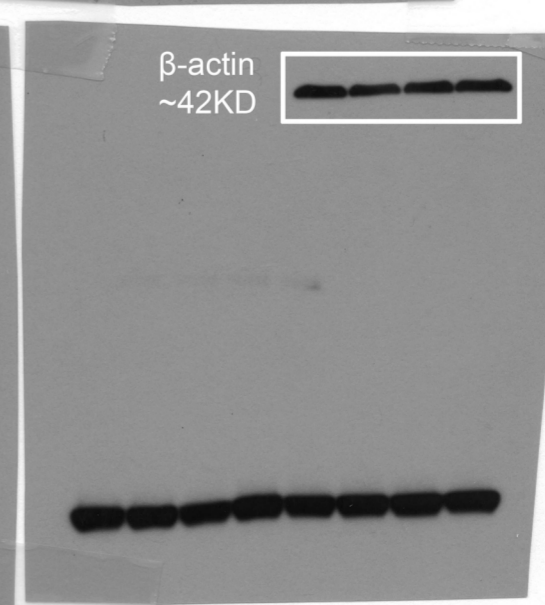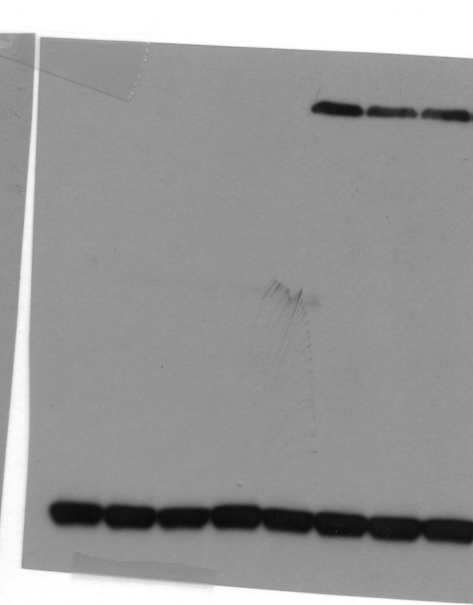

21861-010-050 80 90504

FOXO1  
~78KD

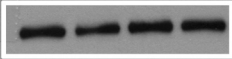

pAKT  
~60KD

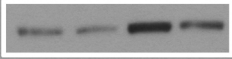

pFOXO1  
~78KD

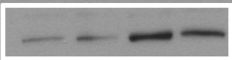

101 5 4 2 105 5 3 4

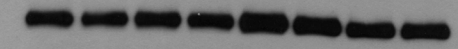

FOXO1

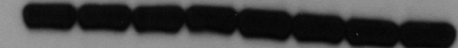

AKT

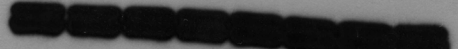

ERK

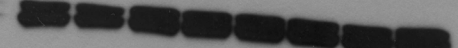

pERK

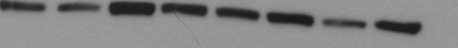

pAKT

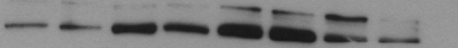

pFOXO1

AKT  
~60KD

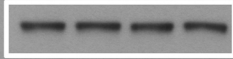

ERK  
~42/44KD

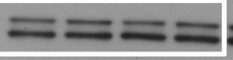

pERK  
~42/44KD

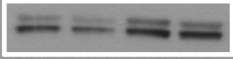

GAPDH  
~36KD

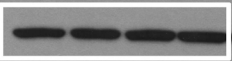

Ab9202

Supplement: SourceData F9 — is the source file for Fig. 9. [file jem_20241799_sourcedataf9.pdf]
